# Supplementary material for: Physiactisome: A New Nanovesicle Drug Containing Heat Shock Protein 60 for Treating Muscle Wasting and Cachexia
Source: Cells. 2022 Apr 21;11(9):1406. doi: 10.3390/cells11091406 (PMC9100106; doi:10.3390/cells11091406)
Supplement: Supplementary file 1 [file cells-11-01406-s001.zip › Supporting_Information_Materials and Methods_Cells_Rev 2.pdf]

SUPPLEMENTARY INFORMATION MATERIALS AND METHODS

Cell culture

C2C12 cells (mouse C3H muscle myoblasts, Cat. no. 91031101, ATCC: CRL1772, Sigma-Aldrich–Merck, Saint Louis, MO, USA), were grown in high glucose Dulbecco's modified Eagle's medium supplemented with 2 mM glutamine and sodium pyruvate (DMEM, Cat. no. 41966052, Gibco–Thermo Fisher Scientific, Waltham, MA, USA), 15% foetal bovine serum (ultracentrifuged, Cat. no. 16000044, Gibco–Thermo Fisher Scientific), and penicillin and streptomycin (Antibiotic-Antimycotic solution, Cat. no. 15240062, Gibco–Thermo Fisher Scientific) at 37°C in a humidified atmosphere with 5% CO_2. Semi-confluent cultures were split 1:3 to 1:6, i.e., seeding at 1–2 × 1,000 cells/cm^2 using 0.25% Trypsin in Dulbecco phosphate-buffered saline without calcium and magnesium (DPBS, Cat. no. 14190094, Gibco–Thermo Fisher Scientific) at 5% CO_2 and 37°C. The cells were not allowed to reach confluence. For immunofluorescence, after 24 h, the cells were fixed in icy methanol and were lysed directly in wells with 500 µL of Tri Reagent (Cat. no. 93289, Sigma-Aldrich–Merck,) for RNA extraction.

Plasmid and bacterial transformation

The plasmid pCMV-6-Entry vector containing the *HSPD1* gene (pCMV-6-Entry-HSPD1, Cat. no. MR222671, OriGene, Rockville, MD, USA) was used to overexpress the protein Hsp60. The resulting protein was a fusion-protein between Hsp60 and the Myc-DDK tag (Hspd1-Myc-DDK-tagged). The sequence of the entire plasmid is shown below.
The HSPD1 sequence used to create the vector was taken from the National Center for Biotechnology Information database (accession number NM_010477), in. For further details of the sequences, see Supporting Information.
The plasmids were amplified following transformation into XL10-Gold ultra-competent bacterial cells (Cat. no. 200315, Agilent technologies, Santa Clara, CA, USA), which are deficient in all known restriction systems [D(mcrA)183 D(mcrCB-hsdSMR-mrr)173] and in endonuclease (endA), in accordance with the manufacturer's instructions.
An aliquot of 100 µL of cells was gently mixed, in a pre-chilled tube, with 4 µL of β-mercaptoethanol provided by the manufacturer. Bacteria incubated on ice for 10 min were swirled gently every 2 min, 50 ng of p-CMV-6-Entry-HSPD1 or the negative control (pCMV6-Entry, mammalian vector with C-terminal Myc- DDK Tag, MOCK, pCMV-6-Entry vector, Cat. no. PS100001, Origene) was then added to the bacterial aliquots.
The vials were incubated on ice for 30 min, after which bacteria underwent a heat shock, a pulse at 42°C for 30 s, followed by an incubation on ice for 2 min. After this incubation, 0.9 mL of preheated (42°C) LB broth (L3397, Sigma, Aldrich–Merck) was added to each vial, and they were incubated at 37°C for 1 h with shaking at 225–250 rpm.
Two-hundred microliters of the transformation mixture was then plated onto LB agar plates containing 50 µg/mL Kanamycin (Cat. no. 11815-024, Thermo Fisher Scientific) and incubated at 37°C overnight. Overnight colonies were used to inoculate 200 mL of LB broth supplemented with 50 µg/mL Kanamycin, and the cultures were incubated overnight at 37°C with shaking. From this bacterial culture, plasmid DNA was extracted using the Qiagen Plasmid Maxi Kit (Cat. no. 12163, Qiagen, Hilden, Germany), following the manufacturer's instructions. The DNA pellet was washed two times with 70% ethanol and dissolved in 500 µL of the TE buffer provided, according to the manufacturer's protocol. DNA was quantified using a NanoDrop ND-2000 (NanoDrop, Thermo Fisher Scientific).

Preparation of Physiactisome

C2C12 cells were transfected with the two plasmids, pCMV6-Entry (pCMV6-Entry Mammalian Expression Vector, Cat. no.: PS100001, OriGene Technologies, Rockville, MD, USA) and pCMV6-

Entry-HSPD1 (Hspd1 (NM_010477) Mouse Tagged ORF Clone, Cat. no.: MR222671, OriGene Technologies), using the Electroporator Neon Transfection System (Thermo Fisher Scientific) and 100 μL of the Neon Transfection System (Cat. no. MPK10025, Thermo Fisher Scientific) according to the manufacturer's instructions.
Briefly, one day before transfection, the cells were plated into 6-well dishes (5×10^4 cell/well).
On the day of the transfection, the cells were 70–90% confluent. The cells were harvested with DPBS without calcium and magnesium and 10% Trypsin/EDTA (Cat. no. 15400054, Gibco – Thermo Fisher Scientific). For each transfection there were 60,000 cells, 36 μg of concentrated plasmid DNA, and 100 μL of Buffer R. The pulse voltage was 1650 V with a 10 ms pulse width, and a total of two pulses were used, according to the manufacturer's instructions. After electroporation, the cells were plated into 6-well dishes with DMEM complete medium without antibiotics/antimycotics.
Two days after transfection, the cells were plated into chamber slides for confocal analysis (5,000 cells/well) and into 6-well plates (50,000 cells/well) to collect the medium and analyse it for the presence of extracellular nanovesicles.

Transmission electron microscopy in 3D cultures
A three-dimensional culture in collagen gel was used to visualise small and large EVs, because this method allows for a non-invasive assessment of whether the EVs are released from cells.
After 3 days of differentiation in DMEM enriched in 5% horse serum (Cat. no. 16050130, Gibco–Thermo Fisher Scientific) as previously described [27], the cells were cultured and embedded onto collagen I (rat tail, Cat. no. 354236, BD Biosciences, Franklin Lakes, NJ, USA) on 24-well plate inserts (200,000 cells/insert; BD Biosciences). After 24 h, the inserts were fixed in 4% PFA for 6 h and then washed in PBS overnight. After two rapid washes in PBS, the inserts were dehydrated in increasing concentrations of ethanol (30%, 50%, and 70%) and then embedded into liquid LR-White Medium Grade Resin (Cat. no. 14380, Electron Microscopy Sciences, Hatfield, PA, USA) before inclusion into gelatin capsules (Cat. no. 70115, Electron Microscopy Sciences). Samples were then cut into 70 nm-thick ultrathin sections, layered onto Formvar-coated gold grids (FCF100-Au-50, Electron Microscopy Sciences), and immunogold labelled.
For staining, the grids were rinsed with water drops for 10 min, incubated in citrate buffer pH 6.0 for 40 min, and blocked in 3% BSA-c (Cat. no. 900099, Electron Microscopy Sciences) in T-PBS for 30 min; they were then incubated with the primary antibody (anti-Alix, 1A12, sc-53540, Santa Cruz Biotechnology, Dallas, TX, USA, diluted 1:10) overnight at 4°C inside a microplate, washed two times for 5 min in T-PBS, and incubated with the AuroProbe secondary antibody (RPN425, Amersham Biosciences, Little Chalfont, UK, diluted 1:25 in 0.3% BSA-c in T-PBS) for 1 h. The grids, washed twice in T-PBS, were then post-fixed with 2% glutaraldehyde in PBS. The grids were contrasted using conventional techniques. Grid-mounted preparations were stained with uranyl acetate and lead citrate for 5 min, and subsequently observed under the JEM-1220 (Jeol) electron microscope examined at 120 kV.

Isolation of C2C12 extracellular vesicles
The medium was first centrifuged at 300 × *g* for 10 min at 4°C to remove the cells; a second centrifugation was performed at 2,000 × *g* for 10 min at 4°C to remove dead cells; then, the medium was centrifuged at 15,000 × *g* for 45 min at 4°C to eliminate cell debris and microparticles. The resulting pellet (large EVs) was stored at –80°C for further analysis, while the remaining supernatant was finally ultracentrifuged at 110,000 × *g* for 2 h at 4°C (small EVs) (Sorvall Discovery Micro-Ultracentrifuge M120 SE ultracentrifuge, type S55A-Ti rotor, Sorvall, Thermo Fischer Scientific). After the first

ultracentrifugation, the pellet was washed with cold PBS and ultracentrifuged again at 110,000 × *g* for 2 h at 4°C. At the end of this step, the supernatant was discarded, and the pellet (EVs) was resuspended in 80 µL of RIPA buffer and quantified using a BCA protein assay (Cat. no. 23225, Thermo Fisher Scientific).

Recombinant HSP60

An oligo corresponding to the sequence coding for human Hsp60 but lacking the sequence required for mitochondrial localisation [Homo sapiens heat shock protein family D (Hsp60) member 1 (HSPD1), transcript variant 1 mRNA, taken from GenBank Accession number NM_002156, 1 to 1655 bp], was synthesised, amplified, and inserted into the pET15b expression plasmid (Eurofins Scientific, Luxembourg) between the sequences for the restriction enzymes BamH1 at the 3′ end and Ndel at the 5′ end. The plasmid allows for the synthesis of a protein with a His-tag at the amino-terminal end. BL21 (DE3) bacteria (Cat. No. 200131, Agilent Technologies) were used for protein expression. The transformed bacteria were grown on LB broth soil plates (L3397, Sigma-Aldrich–Merck) containing agar (Cat. No. A5306, Sigma-Aldrich–Merck) and ampicillin (Cat. No. A9518, Sigma-Aldrich–Merck), at a final concentration of 100 µg/mL, for about 16 h at 37°C. Twenty millilitres of LB broth containing 0.5% glucose (Cat. No. 49163, Sigma-Aldrich – Merck) and ampicillin 100 µg/mL was used to prepare a pre-inoculum of a chosen colony. This pre-inoculum was allowed to grow overnight at 37°C before being added to 1 L of LB broth containing 0.5% glucose and 100 µg/mL ampicillin. The culture was agitated at 37°C until it reached an OD_{600} between 0.5 and 0.6. The expression of HSP60 was induced by the addition of 1 mM isopropyl-β-D-1-thiogalactopyranoside (IPTG, Cat. No. I6758, Sigma-Aldrich–Merck) and the bacterial culture was left to grow at 37°C for 2 h with shaking. After centrifugation at 8,000 rpm for 30 min, the bacterial pellet was resuspended in 30 mL of 50 mM Tris-HCl pH 8, 2 mM threo-1,4-dimercapto-2,3-butanediol, DL-dithiothreitol (DTT, Cat. No. DTT-RO, Sigma-Aldrich–Merck), 2 mM DNAse I (RQ1 RNase-Free DNase, M6101, Promega, Madison, WI, USA), and 10 mM $MgCl_2$ and lysed by sonication. The recombinant protein was then purified using chromatography (AKTA Pure chromatography system, GE Healthcare, Chicago, IL, USA) on column Q sepharose 26/20 using the following buffers: A) 50 mM Tris-HCl pH 8, 2 mM DTT and B) 50 mM Tris-HCl pH 8, 2 mM DTT, 1 M NaCl. After visualisation on an agarose gel, fractions containing the protein were subjected to gel filtration using a Superdex-200 16/60 column with a buffer containing 20 mM Tris-HCl pH 8, 300 mM NaCl, and 0.1 mM Tris (2- carboxyethyl) phosphine (TCEP). The protein was further filtered using centrifuge filters with a cut-off of 30 kDa (Millipore Amicon Ultra 4) to obtain the recombinant protein in a solution containing 20 mM Tris-HCl (pH 7.7), 3% glycerol, and 30 mM NaCl. The protein concentration was obtained by spectrophotometry. The sequence of Hsp60 from 1 to 1,655 nucleotides, from which the recombinant human Hsp60 was obtained, is shown in the Supporting Information.

Liquid chromatography and tandem mass spectrometry

Mass spectrometry (MS) data were acquired using an Orbitrap Fusion Tribrid (Q-OT-qIT) mass spectrometer (Thermo Fisher Scientific) equipped with a Thermo Fisher Scientific Dionex UltiMate 3000 RSLCnano system (Sunnyvale, CA, USA). A 1 µL aliquot of the in-solution digestion was loaded onto an Acclaim Nano Trap C18 column (100 µm i.d. × 2 cm, 5 µm particle size, 100Å). After washing the trapping column with solvent A (H_2O + 0.1% FA) for 3 min at a flow rate of 7 µL/min, peptides were eluted from the trapping column onto a PepMap RSLC C18 EASY-Spray, 75 µm × 50 cm, 2 µm,

100 Å column and were separated by elution at a flow rate of 0.25 µL/min at 40°C, with a linear gradient of solvent B (CH_3CN + 0.1% FA) in A, 5% for 3 min, followed by 5% to 20% in 32 min, 20% to 40% in 30 min, 40% to 60% in 20 min, and 60% to 98% in 15 min.
The eluted peptides were ionised by a nanospray (Easy-spray ion source, Thermo Fisher Scientific) using a spray voltage of 1.7 kV and introduced into the mass spectrometer through a heated ion transfer tube (275°C). Survey scans of peptide precursors in the m/z range 400–1,600 were performed at a resolution of 120,000 (@ 200 m/z) with an AGC target for Orbitrap survey of 4.0×10^5 and a maximum injection time of 50 ms. Tandem MS was performed by isolation at 1.6 Th with the quadrupole, and high-energy collisional dissociation was performed in the ion routing multipole, using a normalised collision energy of 35 and rapid scan MS analysis in the ion trap. Only precursors with charge state 2–4 and an intensity above the threshold of 5×10^3 were sampled for MS2. The dynamic exclusion duration was set to 60 s with a 10 ppm tolerance around the selected precursor and its isotopes. Monoisotopic precursor selection mode was turned on. AGC target and maximum injection time (ms) for tandem MS (MS/MS) spectra were 10,000 and 100, respectively. The instrument was run in top speed mode with 3 s cycles, which means that the instrument continuously performed MS2 events until the list of non-excluded precursors diminished to zero or 3 s, whichever occurred earlier. MS/MS spectral quality was enhanced enabling the parallelisable time option (i.e. by using all parallelisable time during full scan detection for MS/MS precursor injection and detection). Each sample was injected in triplicate to assess the reproducibility of the MS data. MS calibration was performed using the Pierce LTQ Velos ESI Positive Ion Calibration Solution (Thermo Fisher Scientific). MS data acquisition was performed using the Xcalibur v. 3.0.63 software (Thermo Fisher Scientific).

Database searches, protein identification, and label-free quantification analysis

LC–MS/MS data were processed using PEAKS software v. X (Bioinformatics Solutions, Waterloo, ON, Canada). The data were searched against the 17449 "Mus musculus" SwissProt database (release February 2019) to which the yeast enolase 1 (P00924) sequence was added. Tryptic peptides with a maximum of three missed cleavage sites were subjected to an *in silico* search. Cysteine carboxyamidomethylation was set as a fixed modification, whereas oxidation of methionine and transformation of N-terminal glutamine and N-terminal glutamic acid residues in the form of pyroglutamic acid were included as variable modifications. The precursor mass tolerance threshold was 10 ppm, and the maximum fragment mass error was set to 0.6 Da. Peptide spectral matches (PSM) were validated using the Target Decoy PSM Validator node based on q-values at a 1% false discovery rate. Only the proteins identified in at least two replicates with a minimum of two peptides matched were considered.
Proteins containing the same peptides, which could not be differentiated based on MS/MS analysis alone were grouped to satisfy the principles of parsimony.
Label-free quantification data were obtained using PEAKS Q software, which detected the reference sample and automatically aligned the sample runs. Enolase tryptic peptides internal standards were employed as a normalisation factor. Proteins present at distinctly different levels between the two samples were identified by a statistical analysis tool set with the following filters: protein fold change ≥ 2, protein significance ≥ 20, unique peptides ≥ 2, average area < 1.104, and P value < 0.005 The data were displayed in a volcano plot format for ready visualisation.
The MS proteomics data have been deposited to the ProteomeXchange Consortium via the PRIDE partner repository with the dataset identifier PXD017395.

pCMV-6-Entry-HSPD1 vector complete sequence

To overexpress the protein Hsp60 it has been used the plasmid pCMV-6-Entry vector with the HSPD1 gene (pCMV-6-Entry-HSPD1, cat. n. MR222671, Origene Inc., Rockville, Maryland, USA).

Complete sequence of the vector plus insert:
>MR222671 The complete (insert plus vector) sequence of [MR222671] clone

AACAAAATATTAACGCTTACAATTTCCATTCGCCATTCAGGCTGCGCAACTGTTGG
GAAGGGCGATCGGTGCGGGCCTCTTCGCTATTACGCCAGCTGGCGAAAGGGGGATGTGC
TGCAAGGCGATTAAGTTGGGTAACGCCAGGGTTTTCCCAGTCACGACGTTGTAAAACGA
CGGCCAGTGCCAAGCTGATCTATACATTGAATCAATATTGGCAATTAGCCATATTAGTCA
TTGGTTATATAGCATAAATCAATATTGGCTATTGGCCATTGCATACGTTGTATCTATATCA
TAATATGTACATTTATATTGGCTCATGTCCAATATGACCGCCATGTTGACATTGATTATTG
ACTAGTTATTAATAGTAATCAATTACGGGGTCATTAGTTCATAGCCCATATATGGAGTTC
CGCGTTACATAACTTACGGTAAATGGCCCGCCTGGCTGACCGCCCAACGACCCCCGCCCA
TTGACGTCAATAATGACGTATGTTCCCATAGTAACGCCAATAGGGACTTTCCATTGACGT
CAATGGGTGGAGTATTTACGGTAAACTGCCCACTTGGCAGTACATCAAGTGTATCATATG
CCAAGTCCGCCCCCTATTGACGTCAATGACGGTAAATGGCCCGCCTGGCATTATGCCCAG
TACATGACCTTACGGGACTTTCCTACTTGGCAGTACATCTACGTATTAGTCATCGCTATTA
CCATGGTGATGCGGTTTTGGCAGTACACCAATGGGCGTGGATAGCGGTTTGACTCACGGG
GATTTCCAAGTCTCCACCCCATTGACGTCAATGGGAGTTTGTTTTGGCACCAAAATCAAC
GGGACTTTCCAAAATGTCGTAATAACCCCGCCCCGTTGACGCAAATGGGCGGTAGGCGT
GTACGGTGGGAGGTCTATATAAGCAGAGCTCGTTTAGTGAACCGTCAGAATTTTGTAATA
CGACTCACTATAGGGCGGCCGGGAATTCGTCGACTGGATCCGGTACCGAGGAGATCTGC
CGCCGCGATCGCCATGCTTCGACTACCCACAGTCCTTCGCCAGATGAGACCAGTGTCCCG
GGCACTGGCTCCTCATCTCACTCGGGCCTATGCCAAAGATGTAAAATTTGGTGCGGACGC
TCGAGCCTTAATGCTTCAAGGTGTAGACCTTTTAGCAGATGCTGTAGCTGTTACAATGGG
GCCAAAGGGAAGAACTGTGATTATTGAACAGAGTTGGGGAAGTCCCAAAGTAACAAAA
GATGGGGTCACTGTTGCAAAGTCAATTGATTTAAAGGATAAATACAAAAATATTGGAGC
TAAACTTGTTCAGGACGTTGCCAATAACACAAACGAAGAGGCTGGGGATGGCACCACCA
CTGCCACTGTTCTGGCACGATCTATTGCCAAGGAGGGCTTTGAGAAGATCAGCAAAGGG
GCTAATCCAGTGGAAATCCGGAGAGGTGTGATGTTGGCTGTGGATGCTGTAATTGCTGAA
CTTAAGAAACAGTCTAAACCTGTGACAACCCCTGAAGAAATTGCTCAGGTTGCTACAATT
TCTGCAAATGGAGACAAAGACATTGGGAACATCATTTCTGATGCAATGAAAAAGGTTGG
AAGAAAGGGTGTCATCACAGTGAAGGATGGAAAAACCCTGAATGATGAGCTAGAAATTA
TTGAAGGCATGAAGTTTGATAGAGGATATATTTCCCCGTATTTTATTAACACATCAAAAG
GTCAAAAGTGTGAATTCCAAGATGCCTATGTCTTGTTGAGTGAAAAGAAAATTTCCAGTG
TTCAGTCCATTGTCCCTGCTCTTGAAATTGCTAATGCTCATCGGAAGCCATTGGTCATAAT
CGCCGAAGACGTTGACGGAGAAGCTCTAAGCACGCTGGTTTTGAACAGGCTAAAAGTTG
GTCTTCAGGTTGTGGCAGTCAAAGCTCCAGGATTTGGGGACAATAGGAAGAACCAGCTT
AAAGATATGGCTATTGCTACTGGTGGTGCAGTGTTTGGAGAAGAGGGGTTGAATCTAAA
TCTTGAAGATGTTCAAGCTCATGACTTAGGAAAAGTTGGGGAGGTCATTGTCACCAAAG
ATGATGCCATGCTTTTGAAAGGAAAAGGTGACAAAGCTCACATTGAAAAACGTATTCAA
GAAATCACTGAGCAGCTAGACATCACAACTAGTGAATATGAAAAAGAAAAGCTGAACG
AGCGACTTGCTAAACTTTCAGATGGAGTAGCTGTGTTGAAGGTTGGAGGAACAAGTGAT

GTTGAAGTGAATGAGAAAAAAGACAGAGTTACTGATGCTCTCAATGCTACAAGAGCAGC
TGTTGAAGAAGGCATTGTTCTAGGAGGGGGCTGCGCTCTGCTTCGGTGCATCCCAGCCTT
GGATTCATTAAAGCCTGCTAATGAAGACCAGAAAATAGGTATAGAAATTATTAAAAGAG
CACTTAAAATTCCTGCAATGACGATTGCTAAGAATGCAGGTGTTGAAGGATCTTTGATAG
TTGAGAAAATTCTGCAGAGTTCCTCAGAAGTTGGTTATGACGCCATGCTTGGAGATTTTG
TGAACATGGTGGAAAAAGGGATCATTGATCCAACAAAGGTTGTGAGAACTGCCTTACTG
GATGCTGCTGGGGTGGCCTCCTTGCTAACTACAGCCGAAGCTGTAGTGACAGAAATTCCT
AAAGAAGAGAAGGACCCTGGAATGGGTGCAATGGGTGGCATGGGAGGGGGTATGGGAG
GCGGCATGTTCACGCGTACGCGGCCGCTCGAGCAGAAACTCATCTCAGAAGAGGATCTG
GCAGCAAATGATATCCTGGATTACAAGGATGACGACGATAAGGTTTAAACGGCCGGCCG
CGGTCATAGCTGTTTCCTGAACAGATCCCGGGTGGCATCCCTGTGACCCCTCCCCAGTGC
CTCTCCTGGCCCTGGAAGTTGCCACTCCAGTGCCCACCAGCCTTGTCCTAATAAAATTAA
GTTGCATCATTTTGTCTGACTAGGTGTCCTTCTATAATATTATGGGGTGGAGGGGGGTGG
TATGGAGCAAGGGGCAAGTTGGGAAGACAACCTGTAGGGCCTGCGGGGTCTATTGGGAA
CCAAGCTGGAGTGCAGTGGCACAATCTTGGCTCACTGCAATCTCCGCCTCCTGGGTTCAA
GCGATTCTCCTGCCTCAGCCTCCCGAGTTGTTGGGATTCCAGGCATGCATGACCAGGCTC
AGCTAATTTTTGTTTTTTTGGTAGAGACGGGGTTTCACCATATTGGCCAGGCTGGTCTCCA
ACTCCTAATCTCAGGTGATCTACCCACCTTGGCCTCCCAAATTGCTGGGATTACAGGCGT
GAACCACTGCTCCCTTCCCTGTCCTTCTGATTTTAAAATAACTATACCAGCAGGAGGACG
TCCAGACACAGCATAGGCTACCTGGCCATGCCCAACCGGTGGGACATTTGAGTTGCTTGC
TTGGCACTGTCCTCTCATGCGTTGGGTCCACTCAGTAGATGCCTGTTGAATTGGGTACGC
GGCCAGCGGCGAGCGGTATCAGCTCACTCAAAGGCGGTAATACGGTTATCCACAGAATC
AGGGGATAACGCAGGAAAGAACATGTGAGCAAAAGGCCAGCAAAAGGCCAGGAACCGT
AAAAAGGCCGCGTTGCTGGCGTTTTTCCATAGGCTCCGCCCCCCTGACGAGCATCACAAA
AATCGACGCTCAAGTCAGAGGTGGCGAAACCCGACAGGACTATAAAGATACCAGGCGTT
TCCCCCTGGAAGCTCCCTCGTGCGCTCTCCTGTTCCGACCCTGCCGCTTACCGGATACCTG
TCCGCCTTTCTCCCTTCGGGAAGCGTGGCGCTTTCTCATAGCTCACGCTGTAGGTATCTCA
GTTCGGTGTAGGTCGTTCGCTCCAAGCTGGGCTGTGTGCACGAACCCCCCGTTCAGCCCG
ACCGCTGCGCCTTATCCGGTAACTATCGTCTTGAGTCCAACCCGGTAAGACACGACTTAT
CGCCACTGGCAGCAGCCACTGGTAACAGGATTAGCAGAGCGAGGTATGTAGGCGGTGCT
ACAGAGTTCTTGAAGTGGTGGCCTAACTACGGCTACACTAGAAGAACAGTATTTGGTATC
TGCGCTCTGCTGAAGCCAGTTACCTTCGGAAAAAGAGTTGGTAGCTCTTGATCCGGCAAA
CAAACCACCGCTGGTAGCGGTGGTTTTTTTGTTTGCAAGCAGCAGATTACGCGCAGAAAA
AAAGGATCTCAAGAAGATCCTTTGATCTTTTCTACGGGGTCTGACGCTCAGTGGAACGAA
AACTCACGTTAAGGGATTTTGGTCATGAGATTATCAAAAAGGATCTTCACCTAGATCCTT
TTAAATTAAAAATGAAGTTTTAAATCAATCTAAAGTATATATGAGTAACCTGAGGCTATG
GCAGGGCCTGCCGCCCCGACGTTGGCTGCGAGCCCTGGGCCTTCACCCGAACTTGGGGG
GTGGGGTGGGGAAAAGGAAGAAACGCGGGCGTATTGGCCCCAATGGGGTCTCGGTGGG
GTATCGACAGAGTGCCAGCCCTGGGACCGAACCCCGCGTTTATGAACAAACGACCCAAC
ACCGTGCGTTTTATTCTGTCTTTTTATTGCCGTCATAGCGCGGGTTCCTTCCGGTATTGTCT
CCTTCCGTGTTTCAGTTAGCCTCCCCCTAGGGTGGGCGAAGAACTCCAGCATGAGATCCC
CGCGCTGGAGGATCATCCAGCCGGCGTCCCGGAAAACGATTCCGAAGCCCAACCTTTCA
TAGAAGGCGGCGGTGGAATCGAAATCTCGTGATGGCAGGTTGGGCGTCGCTTGGTCGGT
CATTTCGAACCCCAGAGTCCCGCTCAGAAGAACTCGTCAAGAAGGCGATAGAAGGCGAT
GCGCTGCGAATCGGGAGCGGCGATACCGTAAAGCACGAGGAAGCGGTCAGCCCATTCGC
CGCCAAGCTCTTCAGCAATATCACGGGTAGCCAACGCTATGTCCTGATAGCGATCCGCCA
CACCCAGCCGGCCACAGTCGATGAATCCAGAAAAGCGGCCATTTTCCACCATGATATTCG
GCAAGCAGGCATCGCCATGGGTCACGACGAGATCCTCGCCGTCGGGCATGCTCGCCTTG

AGCCTGGCGAACAGTTCGGCTGGCGCGAGCCCCTGATGCTCTTCGTCCAGATCATCCTGA
TCGACAAGACCGGCTTCCATCCGAGTACGTGCTCGCTCGATGCGATGTTTCGCTTGGTGG
TCGAATGGGCAGGTAGCCGGATCAAGCGTATGCAGCCGCCGCATTGCATCAGCCATGAT
GGATACTTTCTCGGCAGGAGCAAGGTGAGATGACAGGAGATCCTGCCCCGGCACTTCGC
CCAATAGCAGCCAGTCCCTTCCCGCTTCAGTGACAACGTCGAGCACAGCTGCGCAAGGA
ACGCCCGTCGTGGCCAGCCACGATAGCCGCGCTGCCTCGTCTTGCAGTTCATTCAGGGCA
CCGGACAGGTCGGTCTTGACAAAAAGAACCGGGCGCCCCTGCGCTGACAGCCGGAACAC
GGCGGCATCAGAGCAGCCGATTGTCTGTTGTGCCCAGTCATAGCCGAATAGCCTCTCCAC
CCAAGCGGCCGGAGAACCTGCGTGCAATCCATCTTGTTCAATCATGCGAAACGATCCTCA
TCCTGTCTCTTGATCGATCTTTGCAAAAGCCTAGGCCTCCAAAAAAGCCTCCTCACTACTT
CTGGAATAGCTCAGAGGCCGAGGCGGCCTCGGCCTCTGCATAAATAAAAAAAATTAGTC
AGCCATGGGGCGGAGAATGGGCGGAACTGGGCGGAGTTAGGGGCGGGATGGGCGGAGT
TAGGGGCGGGACTATGGTTGCTGACTAATTGAGATGCATGCTTTGCATACTTCTGCCTGC
TGGGGAGCCTGGGGACTTTCCACACCTGGTTGCTGACTAATTGAGATGCATGCTTTGCAT
ACTTCTGCCTGCTGGGGAGCCTGGGGACTTTCCACACCCTAACTGACACACATTCCACAG
CTGGTTCTTTCCGCCTCAGGACTCTTCCTTTTTCAATATTATTGAAGCATTTATCAGGGTT
ATTGTCTCATGAGCGGATACATATTTGAATGTATTTAGAAAAATAAACAAATAGGGGTTC
CGCGCACATTTCCCCGAAAAGTGCCACCTGACGCGCCCTGTAGCGGCGCATTAAGCGCG
GCGGGTGTGGTGGTTACGCGCAGCGTGACCGCTACACTTGCCAGCGCCCTAGCGCCCGCT
CCTTTCGCTTTCTTCCCTTCCTTTCTCGCCACGTTCGCCGGCTTTCCCCGTCAAGCTCTAAA
TCGGGGGCTCCCTTTAGGGTTCCGATTTAGTGCTTTACGGCACCTCGACCCCAAAAAACT
TGATTAGGGTGATGGTTCACGTAGTGGGCCATCGCCCTGATAGACGGTTTTTCGCCCTTT
GACGTTGGAGTCCACGTTCTTTAATAGTGGACTCTTGTTCCAAACTGGAACAACACTCAA
CCCTATCTCGGTCTATTCTTTTGATTTATAAGGGATTTTGCCGATTTCGGCCTATTGGTTA
AAAAATGAGCTGATTTAACAAAAATTTAACGCGAATTTT

In black the vector backbone, in red the cloning site used by Origene to clone HSPD1 gene, in green the Myc-DDK-tag.

The sequence of the HSPD1 gene and the resulting Hsp60 protein is shown in Fig. 1s. The protein expressed in C2C12 cells had the Myc-DDK tag attached to the carboxy-terminal, but between the last 3 amino acids and the Myc-DDK tag (EQKLISEEDLAANDILDYKDDDDK, in green in Fig. 1s, there were 2 aa (TR, in red in Fig. 1s) that derived from the cloning site, and 4 aa (TRPL, in gray, which derived from the structural sequence of the plasmid, which of fact is still translated by the cells.

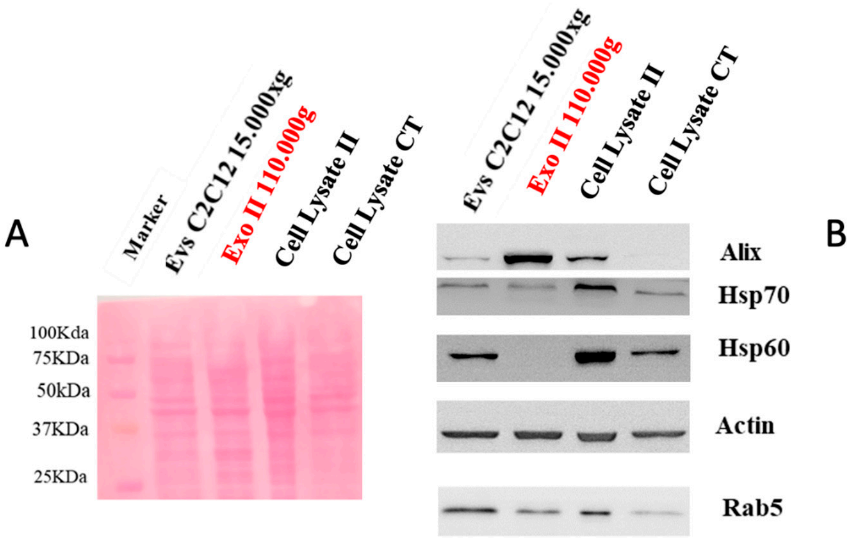

Supplemental Figure S1. Western blot analysis of large extracellular vesicles (EVs), small EVs, and total lysates from C2C12 cells (20 µg of protein per lane), obtained from another experiment, to detect the expression of Alix, HSP70, HSP60 and RAB5. β-Actin was used as a loading control. A - Red Ponceau of Ultracentrifuged Evs, Exosomes and cell Lysate.
B - Western Blot of Ultracentrifuged Evs, Exosomes and Cell Lysate for Alix, Hsp70, Hsp60, Actin and Rab5 antibodies.

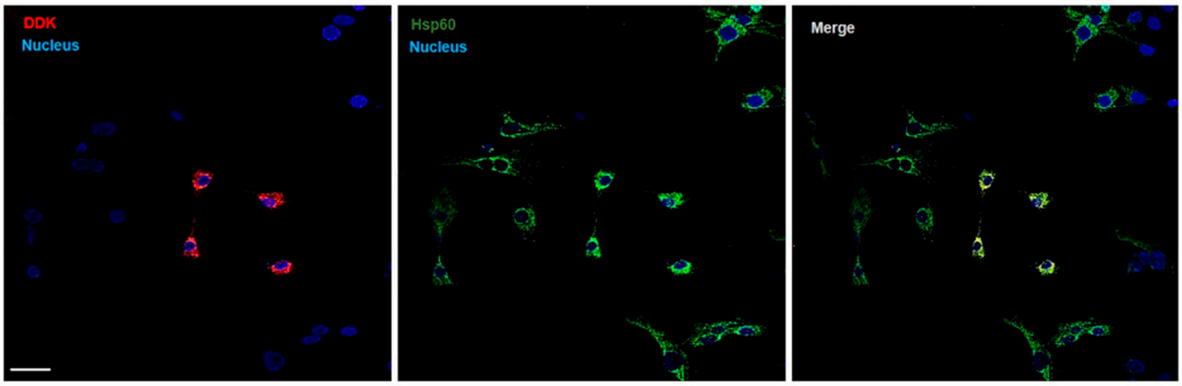

Supplemental Figure S2. Immunofluorescence images of C2C12 myoblasts cell line transfected with pCMV6-Entry-HSPD1 vector showing the presence of Hsp60 within the cells (green). The expression of the tag Myc DDK (red) demonstrates cell transfection. Nuclei were counterstained with DAPI (blue). Bar 50 µm.

27. Romancino, D.P.; Paterniti, G.; Campos, Y.; De Luca, A.; Di Felice, V.; d'Azzo, A.; Bongiovanni, A. Identification and characterization of the nano-sized vesicles released by muscle cells. *FEBS Lett.* **2013**, *587*, 1379–1384.
