# Supplementary material for: Physiactisome: A New Nanovesicle Drug Containing Heat Shock Protein 60 for Treating Muscle Wasting and Cachexia
Source: Cells. 2022 Apr 21;11(9):1406. doi: 10.3390/cells11091406 (PMC9100106; doi:10.3390/cells11091406)
Supplement: Supplementary file 1 [file cells-11-01406-s001.zip › Supporting_Information_Table S1-S4_10_2_2022.pdf]

Table S1. Proteins with differences in relative abundances

Accession	Description	Avg. Mass	Ratio Large:Small EVS	P value
P62806	Histone H4	11367	0.03	1.742E-03
Q64475	Histone H2B type 1-B	13952	0.03	6.848E-06
P11276	Fibronectin	272536	0.05	2.720E-06
O08992	Syntenin-1	32379	0.06	1.201E-05
Q9R045	Angiopoietin-related protein 2	57106	0.08	4.433E-05
Q8BVY0	Ribosomal L1 domain-containing protein 1	50421	0.10	1.857E-07
P11440	Cyclin-dependent kinase 1	34107	0.10	5.098E-04
P68368	Tubulin alpha-4A chain	49924	0.11	7.574E-05
P43276	Histone H1.5 OS=Mus musculus	22576	0.12	1.941E-07
Q61001	Laminin subunit alpha-5	404056	0.12	3.375E-07
Q9WU78	Programmed cell death 6-interacting protein	96024	0.12	4.572E-07
Q8R366	Immunoglobulin superfamily member 8	65011	0.13	1.679E-03
Q61187	Tumor susceptibility gene 101 protein	44124	0.13	7.457E-08
Q9D1C8	Vacuolar protein sorting-associated protein 28 homolog	25452	0.13	5.821E-06
Q9Z204	Heterogeneous nuclear ribonucleoproteins C1/C2	34385	0.14	3.066E-05
Q9QZI8	Serine incorporator 1	50509	0.14	4.400E-05
Q6P5F7	Protein tweety homolog 3	57714	0.14	3.612E-06
E9PYK3	Protein mono-ADP-ribosyltransferase PARP4	216131	0.15	2.014E-04
P62274	40S ribosomal protein S29	6677	0.16	9.961E-05
Q9EQK5	Major vault protein	95924	0.16	7.912E-06
Q8VEK3	Heterogeneous nuclear ribonucleoprotein U	87918	0.16	3.050E-04
Q9WV91	Prostaglandin F2 receptor negative regulator	98722	0.17	9.962E-06
P62281	40S ribosomal protein S11 OS=Mus musculus	18431	0.17	6.823E-06
Q9WVA3	Mitotic checkpoint protein BUB3 OS=Mus musculus	36955	0.17	2.399E-05
P62242	40S ribosomal protein S8 OS=Mus musculus	24205	0.18	6.334E-05
P54775	26S proteasome regulatory subunit 6B	47408	0.18	2.447E-07
Q6ZWU9	40S ribosomal protein S27	9461	0.18	1.026E-05
Q6P4T2	U5 small nuclear ribonucleoprotein 200 kDa helicase	244544	0.18	1.427E-07

P62855	40S ribosomal protein S26 OS=Mus musculus	13015	0.19	1.572E-07
P62196	26S proteasome regulatory subunit 8	45626	0.19	2.083E-03
P62267	40S ribosomal protein S23	15808	0.19	5.737E-05
P62754	40S ribosomal protein S6 OS=Mus musculus	28681	0.19	3.692E-05
Q7TPV4	Myb-binding protein 1A OS=Mus musculus	152036	0.19	5.667E-06
P14131	40S ribosomal protein S16 OS=Mus musculus	16445	0.20	6.201E-06
Q9CZX7	Type 2 phosphatidylinositol 4 5-bisphosphate 4-phosphatase	28038	0.20	1.801E-05
B2RQC6	CAD protein	243236	0.20	1.266E-04
P62192	26S proteasome regulatory subunit 4	49185	0.20	3.937E-04
P25444	40S ribosomal protein S2	31231	0.21	7.067E-06
P46471	26S proteasome regulatory subunit 7	48648	0.21	2.128E-06
P60843	Eukaryotic initiation factor 4A-I	46154	0.21	7.626E-06
P68040	Receptor of activated protein C kinase 1	35077	0.21	4.289E-05
Q3UH60	Disco-interacting protein 2 homolog B	171125	0.22	4.613E-03
O70133	ATP-dependent RNA helicase A	149474	0.22	8.706E-04
P60122	RuvB-like 1	50214	0.22	1.669E-05
P62334	26S proteasome regulatory subunit 10B	44173	0.23	4.754E-05
Q9WTM5	RuvB-like 2	51113	0.23	1.143E-03
Q6ZWN5	40S ribosomal protein S9	22591	0.23	3.767E-06
P62245	40S ribosomal protein S15a	14840	0.23	1.004E-05
O88685	26S proteasome regulatory subunit 6A	49549	0.24	2.170E-06
O08810	116 kDa U5 small nuclear ribonucleoprotein component	109361	0.24	5.256E-04
Q8BH64	EH domain-containing protein 2	61175	0.24	3.728E-05
P62702	40S ribosomal protein S4 X isoform	29598	0.25	1.781E-06
Q6ZWV3	60S ribosomal protein L10	24604	0.25	6.709E-06
P07742	Ribonucleoside-diphosphate reductase large subunit	90210	0.25	2.783E-04
P62908	40S ribosomal protein S3	26674	0.25	2.164E-06
O35286	Pre-mRNA-splicing factor ATP-dependent RNA helicase DHX15	91007	0.25	2.846E-02
P27659	60S ribosomal protein L3	46110	0.25	3.938E-05
Q9EQP2	EH domain-containing protein 4	61481	0.25	3.405E-04
Q9D8E6	60S ribosomal protein L4	47154	0.26	3.064E-05
P32067	Lupus La protein homolog	47756	0.26	1.136E-03

P47964	60S ribosomal protein L36	12216	0.26	3.933E-05
P80316	T-complex protein 1 subunit epsilon	59624	0.26	2.290E-06
O09167	60S ribosomal protein L21	18562	0.27	3.609E-05
Q3UDE2	Tubulin--tyrosine ligase-like protein 12	74043	0.27	7.133E-05
Q99LF4	tRNA-splicing ligase RtcB homolog	55249	0.27	1.065E-04
Q9ERD7	Tubulin beta-3 chain	50419	0.27	4.448E-04
P62264	40S ribosomal protein S14	16273	0.27	3.499E-05
P62717	60S ribosomal protein L18a	20732	0.28	1.618E-06
P62911	60S ribosomal protein L32	15860	0.28	7.856E-05
Q62167	ATP-dependent RNA helicase DDX3X	73102	0.28	1.192E-04
Q921M3	Splicing factor 3B subunit 3	135550	0.28	1.914E-04
Q8VDM4	26S proteasome non-ATPase regulatory subunit 2	100203	0.28	1.480E-05
P16460	Argininosuccinate synthase	46585	0.29	1.216E-06
Q9ESX5	H/ACA ribonucleoprotein complex subunit DKC1	57402	0.29	1.910E-02
P19253	60S ribosomal protein L13a	23464	0.29	3.107E-06
P80317	T-complex protein 1 subunit zeta	58004	0.29	5.022E-05
Q8QZY6	Tetraspanin-14	30674	0.29	4.198E-04
P97351	40S ribosomal protein S3a	29885	0.29	2.089E-07
P42932	T-complex protein 1 subunit theta	59556	0.29	7.448E-06
P62849	40S ribosomal protein S24	15423	0.29	1.865E-04
Q9CR57	60S ribosomal protein L14	23564	0.30	4.723E-04
P14206	40S ribosomal protein SA	32838	0.30	1.140E-05
Q99PV0	Pre-mRNA-processing-splicing factor 8	273615	0.30	8.138E-04
Q9CPR4	60S ribosomal protein L17	21423	0.30	1.204E-04
Q5SUF2	Luc7-like protein 3	51450	0.30	9.372E-05
P62983	Ubiquitin-40S ribosomal protein S27a	17951	0.31	3.124E-06
P11983	T-complex protein 1 subunit alpha	60449	0.31	1.909E-05
P62270	40S ribosomal protein S18	17719	0.31	4.988E-07
P41105	60S ribosomal protein L28	15733	0.32	3.300E-06
Q9CZM2	60S ribosomal protein L15	24146	0.32	7.415E-06
P63037	DnaJ homolog subfamily A member 1	44868	0.32	1.827E-04
P62827	GTP-binding nuclear protein Ran	24423	0.32	5.141E-07

P08030	Adenine phosphoribosyltransferase	19724	0.32	2.674E-04
P30999	Catenin delta-1	104925	0.32	1.111E-03
P80314	T-complex protein 1 subunit beta	57477	0.32	2.186E-06
Q9JIZ9	Phospholipid scramblase 3	31803	0.32	2.876E-06
Q8BP67	60S ribosomal protein L24	17779	0.33	1.414E-05
P68372	Tubulin beta-4B chain	49831	0.33	5.592E-05
Q9CYI4	Putative RNA-binding protein Luc7-like 1	43934	0.33	4.280E-04
P61358	60S ribosomal protein L27	15798	0.34	7.007E-03
Q61656	Probable ATP-dependent RNA helicase DDX5	69290	0.34	2.645E-06
Q9WVK4	EH domain-containing protein 1	60603	0.34	5.190E-04
P62889	60S ribosomal protein L30	12784	0.34	3.217E-05
P47963	60S ribosomal protein L13	24305	0.34	3.442E-06
O35682	Myeloid-associated differentiation marker	35285	0.34	2.774E-04
P60867	40S ribosomal protein S20	13373	0.35	2.748E-05
P62301	40S ribosomal protein S13	17222	0.35	2.750E-04
P12970	60S ribosomal protein L7a	29977	0.35	3.011E-06
P14869	60S acidic ribosomal protein P0	34216	0.35	3.446E-04
P99024	Tubulin beta-5 chain	49671	0.36	3.329E-04
Q8R081	Heterogeneous nuclear ribonucleoprotein L	63964	0.36	7.570E-04
P14115	60S ribosomal protein L27a	16605	0.36	5.060E-03
P62315	Small nuclear ribonucleoprotein Sm D1	13282	0.36	2.943E-04
P14148	60S ribosomal protein L7	31420	0.36	9.123E-04
Q99NB9	Splicing factor 3B subunit 1	145816	0.36	8.207E-04
P84099	60S ribosomal protein L19	23466	0.36	1.762E-05
P23927	Alpha-crystallin B chain	20069	0.36	2.379E-05
P47911	60S ribosomal protein L6	33510	0.36	1.544E-04
Q9Z1Q9	Valine--tRNA ligase	140215	0.36	1.716E-04
P62852	40S ribosomal protein S25	13742	0.37	4.916E-05
O35598	Disintegrin and metalloproteinase domain-containing protein 10	83968	0.37	1.727E-04
O35593	26S proteasome non-ATPase regulatory subunit 14	34577	0.37	8.055E-05
Q9D8W5	26S proteasome non-ATPase regulatory subunit 12	52895	0.37	3.891E-03
Q7TNC4	Putative RNA-binding protein Luc7-like 2	46583	0.37	2.149E-04

P80318	T-complex protein 1 subunit gamma	60630	0.38	6.921E-05
P35979	60S ribosomal protein L12	17805	0.38	5.043E-06
Q8BG32	26S proteasome non-ATPase regulatory subunit 11	47437	0.38	4.579E-06
P62918	60S ribosomal protein L8	28025	0.38	4.967E-06
P12815	Programmed cell death protein 6	21867	0.38	8.601E-04
Q6A0A9	Constitutive coactivator of PPAR-gamma-like protein 1	121646	0.39	1.338E-02
P80315	T-complex protein 1 subunit delta	58066	0.39	2.666E-06
P80313	T-complex protein 1 subunit eta	59652	0.40	1.427E-06
Q9QYJ0	DnaJ homolog subfamily A member 2	45746	0.40	6.173E-04
P97461	40S ribosomal protein S5	22889	0.40	1.465E-06
P23242	Gap junction alpha-1 protein	43004	0.40	4.548E-04
P57784	U2 small nuclear ribonucleoprotein A'	28357	0.40	2.347E-02
Q9CQW9	Interferon-induced transmembrane protein 3	14954	0.40	2.878E-03
Q3TXS7	26S proteasome non-ATPase regulatory subunit 1	105730	0.40	2.968E-05
P62843	40S ribosomal protein S15	17040	0.40	3.602E-05
Q9JHU4	Cytoplasmic dynein 1 heavy chain 1	532050	0.41	1.691E-04
P62137	Serine/threonine-protein phosphatase PP1-alpha catalytic subunit	37540	0.41	1.519E-03
P12382	ATP-dependent 6-phosphofructokinase liver type	85360	0.41	1.939E-03
P63276	40S ribosomal protein S17	15524	0.43	2.775E-05
P06869	Urokinase-type plasminogen activator	48268	0.43	7.282E-05
Q922D8	C-1-tetrahydrofolate synthase cytoplasmic	101200	0.43	2.812E-04
Q8BU30	Isoleucine--tRNA ligase cytoplasmic	144270	0.43	2.277E-03
P26516	26S proteasome non-ATPase regulatory subunit 7	36540	0.43	3.803E-03
Q8BMJ2	Leucine--tRNA ligase cytoplasmic	134192	0.43	1.481E-04
Q8BP47	Asparagine--tRNA ligase cytoplasmic	64279	0.44	2.226E-04
Q9JJ00	Phospholipid scramblase 1	35914	0.44	2.585E-04
Q8CGC7	Bifunctional glutamate/proline--tRNA ligase	170078	0.45	2.811E-04
Q6PDI5	Proteasome adapter and scaffold protein ECM29	203702	0.45	5.396E-03
Q68FL6	Methionine--tRNA ligase cytoplasmic	101431	0.45	1.280E-02
Q9Z1Z2	Serine-threonine kinase receptor-associated protein	38442	0.45	2.417E-04
O35737	Heterogeneous nuclear ribonucleoprotein H	49199	0.46	1.626E-03
P47962	60S ribosomal protein L5	34401	0.46	2.290E-05

P62305	Small nuclear ribonucleoprotein E	10804	0.46	1.723E-03
Q9WVJ2	26S proteasome non-ATPase regulatory subunit 13	42809	0.46	1.303E-02
Q9CXW4	60S ribosomal protein L11	20252	0.46	1.106E-02
P35762	CD81 antigen	25815	0.46	2.123E-03
P40237	CD82 antigen	29629	0.47	1.394E-04
P62830	60S ribosomal protein L23	14865	0.47	1.602E-04
O70194	Eukaryotic translation initiation factor 3 subunit D	63989	0.47	1.142E-03
P28271	Cytoplasmic aconitate hydratase	98126	0.47	2.097E-03
P63325	40S ribosomal protein S10	18916	0.47	4.824E-05
Q8CIE6	Coatomer subunit alpha	138432	0.48	2.115E-04
P35980	60S ribosomal protein L18	21645	0.48	2.743E-02
Q8BY89	Choline transporter-like protein 2	80110	0.50	1.039E-04
P97447	Four and a half LIM domains protein 1	31889	2.00	3.494E-05
Q9EPL8	Importin-7	119486	2.00	1.161E-03
Q9QYB1	Chloride intracellular channel protein 4	28729	2.04	1.918E-05
P11499	Heat shock protein HSP 90-beta	83281	2.04	6.978E-05
Q3U1J4	DNA damage-binding protein 1	126853	2.04	1.216E-03
P62874	Guanine nucleotide-binding protein G(I)/G(S)/G(T) subunit beta-1	37377	2.08	8.512E-05
Q7TMS5	ATP-binding cassette sub-family G member 2	72978	2.08	1.927E-04
Q9DBG3	AP-2 complex subunit beta	104583	2.08	3.511E-04
O54724	Caveolae-associated protein 1	43954	2.08	2.224E-02
Q8BTM8	Filamin-A	281219	2.13	4.941E-05
Q62351	Transferrin receptor protein 1	85731	2.13	1.877E-04
P62962	Profilin-1	14957	2.13	6.624E-04
Q921F2	TAR DNA-binding protein 43	44548	2.13	4.387E-03
P30416	Peptidyl-prolyl cis-trans isomerase FKBP4	51572	2.13	3.727E-02
Q920A5	Retinoid-inducible serine carboxypeptidase	50965	2.17	2.425E-04
Q8BWY3	Eukaryotic peptide chain release factor subunit 1	49031	2.22	1.339E-05
Q75N73	Zinc transporter ZIP14	53962	2.22	1.555E-04
Q91ZJ5	UTP--glucose-1-phosphate uridylyltransferase	56979	2.22	3.949E-04
P26040	Ezrin	69407	2.22	7.972E-04
O88456	Calpain small subunit 1	28463	2.22	2.249E-03

Q64514	Tripeptidyl-peptidase 2	139878	2.22	2.103E-02
Q9WUM3	Coronin-1B	53912	2.27	2.123E-05
Q91YE6	Importin-9	116052	2.27	5.377E-05
P70349	Histidine triad nucleotide-binding protein 1	13777	2.27	1.092E-03
P63024	Vesicle-associated membrane protein 3	11480	2.27	4.234E-03
P62880	Guanine nucleotide-binding protein G(I)/G(S)/G(T) subunit beta-2	37331	2.27	4.562E-03
O35639	Annexin A3	36384	2.33	6.069E-05
P63242	Eukaryotic translation initiation factor 5A-1	16832	2.33	1.284E-04
Q9R0P5	Destrin	18522	2.33	2.071E-04
O54890	Integrin beta-3	86738	2.33	2.494E-04
P43406	Integrin alpha-V	115360	2.33	2.997E-04
P10852	4F2 cell-surface antigen heavy chain	58337	2.33	3.625E-04
Q60634	Flotillin-2	47038	2.33	5.245E-04
Q61171	Peroxiredoxin-2	21779	2.33	1.113E-03
P21278	Guanine nucleotide-binding protein subunit alpha-11	42024	2.33	2.322E-03
Q80UG5	Septin-9	65575	2.33	3.721E-03
P49312	Heterogeneous nuclear ribonucleoprotein A1	34196	2.33	1.584E-02
P35700	Peroxiredoxin-1	22176	2.38	1.999E-05
P58242	Acid sphingomyelinase-like phosphodiesterase 3b	51600	2.38	2.305E-04
P68510	14-3-3 protein eta	28212	2.38	2.339E-04
P13595	Neural cell adhesion molecule 1	119427	2.38	2.145E-03
Q8VBV7	COP9 signalosome complex subunit 8	23256	2.38	5.622E-03
Q8BWM0	Prostaglandin E synthase 2	43324	2.44	1.925E-05
Q61553	Fascin	54508	2.44	2.263E-05
P63028	Translationally-controlled tumor protein	19462	2.44	1.770E-04
P21995	Embigin	37064	2.44	2.355E-04
Q62433	Protein NDRG1	43009	2.44	6.878E-04
P57716	Nicastrin	78492	2.44	3.355E-03
Q11011	Puromycin-sensitive aminopeptidase	103325	2.44	5.727E-03
Q60854	Serpin B6	42599	2.50	9.081E-06
P26041	Moesin	67767	2.50	2.390E-05
P53994	Ras-related protein Rab-2A	23548	2.50	3.492E-05

P07091	Protein S100-A4	11721	2.50	4.775E-05
Q9D819	Inorganic pyrophosphatase	32667	2.50	3.875E-04
Q9CQE8	RNA transcription translation and transport factor protein	28152	2.50	2.325E-02
Q69ZN7	Myoferlin	233322	2.56	1.128E-07
P17809	Solute carrier family 2 facilitated glucose transporter member 1	53985	2.56	8.645E-07
Q62465	Synaptic vesicle membrane protein VAT-1 homolog	43097	2.56	1.032E-05
Q9Z127	Large neutral amino acids transporter small subunit 1	55872	2.56	1.145E-04
P68254	14-3-3 protein theta	27778	2.56	2.129E-04
P18572	Basigin	42445	2.56	4.821E-04
P63321	Ras-related protein Ral-A	23553	2.56	2.618E-03
P28667	MARCKS-related protein	20165	2.63	1.340E-05
P57776	Elongation factor 1-delta	31293	2.63	2.163E-05
Q9R1Q7	Proteolipid protein 2	16608	2.63	8.958E-05
O09117	Synaptophysin-like protein 1	28899	2.63	3.277E-04
P24452	Macrophage-capping protein	39240	2.63	3.993E-04
P50543	Protein S100-A11	11083	2.70	4.118E-06
P63101	14-3-3 protein zeta/delta	27771	2.70	9.602E-06
Q99L47	Hsc70-interacting protein	41656	2.70	1.348E-05
Q61490	CD166 antigen	65092	2.70	9.407E-05
G5E829	Plasma membrane calcium-transporting ATPase 1	134747	2.70	1.613E-04
Q9DCT8	Cysteine-rich protein 2	22727	2.70	6.220E-04
P46061	Ran GTPase-activating protein 1	63531	2.70	7.196E-04
O88792	Junctional adhesion molecule A	32424	2.70	8.849E-03
P16045	Galectin-1	14866	2.78	7.614E-06
P39447	Tight junction protein ZO-1	194740	2.78	1.986E-05
P56399	Ubiquitin carboxyl-terminal hydrolase 5	95833	2.78	3.959E-05
O88342	WD repeat-containing protein 1	66407	2.78	5.138E-05
Q9CQM9	Glutaredoxin-3	37778	2.78	8.721E-05
Q9WVA4	Transgelin-2	22395	2.78	1.103E-04
Q62523	Zyxin	60546	2.78	1.134E-04
P34884	Macrophage migration inhibitory factor	12504	2.78	1.300E-04
Q9CY58	Plasminogen activator inhibitor 1 RNA-binding protein	44714	2.78	1.393E-04

Q8R422	CD109 antigen	161658	2.78	1.621E-04
Q99K51	Plastin-3	70742	2.78	2.194E-04
Q99PT1	Rho GDP-dissociation inhibitor 1	23407	2.78	3.636E-04
Q61598	Rab GDP dissociation inhibitor beta	50537	2.86	8.770E-06
P48036	Annexin A5	35752	2.86	9.324E-06
P53986	Monocarboxylate transporter 1	53267	2.86	1.764E-04
Q61165	Sodium/hydrogen exchanger 1	91468	2.86	5.112E-04
Q99MN1	Lysine--tRNA ligase	67840	2.86	1.354E-03
Q62426	Cystatin-B	11046	2.86	1.467E-02
P17742	Peptidyl-prolyl cis-trans isomerase A	17971	2.94	2.186E-05
P07901	Heat shock protein HSP 90-alpha	84788	2.94	3.340E-05
Q62188	Dihydropyrimidinase-related protein 3	61936	2.94	4.359E-05
P62259	14-3-3 protein epsilon	29174	2.94	5.337E-05
P18242	Cathepsin D	44954	2.94	1.252E-04
Q62261	Spectrin beta chain non-erythrocytic 1	274221	2.94	2.225E-04
Q8C129	Leucyl-cystinyl aminopeptidase	117304	2.94	8.490E-04
P29533	Vascular cell adhesion protein 1	81317	2.94	9.972E-04
P17182	Alpha-enolase	47141	2.94	1.054E-03
P70670	Nascent polypeptide-associated complex subunit alpha muscle-specific form	220497	3.03	7.108E-07
P10639	Thioredoxin	11675	3.03	3.520E-06
Q64727	Vinculin	116717	3.03	7.134E-05
P46638	Ras-related protein Rab-11B	24489	3.03	1.756E-04
Q921M7	Protein FAM49B	36776	3.03	8.879E-04
Q6Q477	Plasma membrane calcium-transporting ATPase 4	133068	3.03	2.018E-03
P0DP26	Calmodulin-1	16838	3.13	2.612E-06
P0DP27	Calmodulin-2	16838	3.13	2.612E-06
P0DP28	Calmodulin-3	16838	3.13	2.612E-06
Q9DBJ1	Phosphoglycerate mutase 1	28832	3.13	2.415E-05
Q8BKC5	Importin-5	123591	3.13	6.257E-05
P17751	Triosephosphate isomerase	32192	3.23	2.350E-05
P18760	Cofilin-1	18560	3.23	2.575E-05

Q8R1F1	Niban-like protein 1	84819	3.23	5.661E-05
Q9D0F9	Phosphoglucomutase-1	61418	3.23	2.530E-04
O08539	Myc box-dependent-interacting protein 1	64470	3.23	3.409E-04
Q6ZQ38	Cullin-associated NEDD8-dissociated protein 1	136331	3.23	9.168E-04
Q8C1A5	Thimet oligopeptidase	78026	3.23	1.153E-03
Q62348	Translin	26201	3.23	1.467E-03
Q9EPR5	VPS10 domain-containing receptor SorCS2	128902	3.23	1.094E-02
Q99020	Heterogeneous nuclear ribonucleoprotein A/B	30831	3.33	2.052E-06
P68037	Ubiquitin-conjugating enzyme E2 L3	17862	3.33	7.410E-05
P0CW02	Lymphocyte antigen 6C1	14192	3.33	1.096E-04
P26645	Myristoylated alanine-rich C-kinase substrate	29661	3.33	1.680E-04
P10107	Annexin A1	38734	3.45	5.041E-08
P40124	Adenylyl cyclase-associated protein 1	51565	3.45	8.866E-05
O08709	Peroxiredoxin-6	24871	3.45	1.411E-04
O88844	Isocitrate dehydrogenase [NADP] cytoplasmic	46674	3.45	2.326E-04
Q05816	Fatty acid-binding protein 5	15137	3.45	1.170E-03
P97300	Neuroplastin	44373	3.57	2.794E-05
P61982	14-3-3 protein gamma	28303	3.57	4.292E-05
Q8VDD8	WASH complex subunit 1	51659	3.57	1.500E-04
Q61316	Heat shock 70 kDa protein 4	94133	3.57	1.532E-04
Q9JM76	Actin-related protein 2/3 complex subunit 3	20525	3.57	1.317E-03
Q80X90	Filamin-B	277822	3.70	2.455E-06
Q9CXW3	Calcyclin-binding protein	26510	3.70	5.959E-06
Q8VDN2	Sodium/potassium-transporting ATPase subunit alpha-1	112982	3.70	1.633E-05
Q9CZU6	Citrate synthase mitochondrial	51737	3.70	2.464E-05
P97370	Sodium/potassium-transporting ATPase subunit beta-3	31776	3.70	8.105E-05
O70251	Elongation factor 1-beta	24694	3.70	9.635E-05
P97315	Cysteine and glycine-rich protein 1	20583	3.70	1.130E-04
P05132	cAMP-dependent protein kinase catalytic subunit alpha	40571	3.70	1.193E-03
Q78ZA7	Nucleosome assembly protein 1-like 4	42679	3.70	4.558E-03
P06801	NADP-dependent malic enzyme	63954	3.70	2.097E-02
Q99LT0	Protein dpy-30 homolog	11213	3.85	4.135E-05

Q920E5	Farnesyl pyrophosphate synthase	40582	3.85	5.873E-05
P06745	Glucose-6-phosphate isomerase	62767	3.85	6.030E-05
Q9CR16	Peptidyl-prolyl cis-trans isomerase D	40743	3.85	5.573E-04
Q9CVB6	Actin-related protein 2/3 complex subunit 2	34357	3.85	3.472E-03
P05064	Fructose-bisphosphate aldolase A	39356	4.00	2.298E-06
P48678	Prelamin-A/C	74238	4.00	7.751E-06
Q99K85	Phosphoserine aminotransferase	40473	4.00	1.600E-05
P06151	L-lactate dehydrogenase A chain	36499	4.00	3.881E-05
P26043	Radixin	68543	4.00	4.216E-05
P97855	Ras GTPase-activating protein-binding protein 1	51829	4.00	9.600E-05
Q9D1D4	Transmembrane emp24 domain-containing protein 10	24911	4.00	2.057E-04
Q64152	Transcription factor BTF3	22031	4.00	4.025E-04
P61087	Ubiquitin-conjugating enzyme E2 K	22407	4.00	1.844E-03
Q60932	Voltage-dependent anion-selective channel protein 1	32351	4.00	3.851E-03
Q60864	Stress-induced-phosphoprotein 1	62582	4.17	1.649E-05
Q9JKF1	Ras GTPase-activating-like protein IQGAP1	188741	4.17	1.892E-05
Q60767	Lymphocyte antigen 75	197351	4.17	5.467E-05
P99029	Peroxiredoxin-5 mitochondrial	21897	4.17	1.111E-04
Q9CPU0	Lactoylglutathione lyase	20810	4.17	9.194E-04
P63280	SUMO-conjugating enzyme UBC9	18007	4.35	2.600E-05
Q8CAY6	Acetyl-CoA acetyltransferase cytosolic	41298	4.35	6.126E-05
O35685	Nuclear migration protein nudC	38358	4.35	6.160E-05
Q9D379	Epoxide hydrolase 1	52577	4.35	6.464E-05
Q60930	Voltage-dependent anion-selective channel protein 2	31733	4.35	4.191E-04
O35379	Multidrug resistance-associated protein 1	171184	4.35	2.756E-03
Q01768	Nucleoside diphosphate kinase B	17363	4.55	1.207E-07
Q9DCD0	6-phosphogluconate dehydrogenase decarboxylating	53247	4.55	1.642E-06
Q9WUM4	Coronin-1C OS=Mus musculus	53121	4.55	1.728E-06
P48999	Arachidonate 5-lipoxygenase	77967	4.55	2.903E-05
O09131	Glutathione S-transferase omega-1	27498	4.55	5.986E-05
Q9DCN2	NADH-cytochrome b5 reductase 3	34128	4.55	9.941E-04
P10518	Delta-aminolevulinic acid dehydratase	36024	4.76	1.926E-06

P45591	Cofilin-2	18710	4.76	1.970E-05
P08228	Superoxide dismutase [Cu-Zn]	15943	4.76	4.914E-05
Q9CXP8	Guanine nucleotide-binding protein G(I)/G(S)/G(O) subunit gamma-10	7229	4.76	1.801E-04
Q6P5H2	Nestin	207122	4.76	3.337E-04
O35640	Annexin A8	36724	5.00	6.747E-05
Q8R5J9	PRA1 family protein 3	21558	5.00	1.989E-04
P14873	Microtubule-associated protein 1B	270252	5.26	1.133E-06
Q9CZN7	Serine hydroxymethyltransferase mitochondrial	55759	5.26	1.281E-03
P42208	Septin-2	41526	5.56	2.119E-04
Q9R1J0	Sterol-4-alpha-carboxylate 3-dehydrogenase decarboxylating	40686	5.56	5.231E-04
Q9JJU8	SH3 domain-binding glutamic acid-rich-like protein	12811	5.56	5.962E-04
Q99P72	Reticulon-4	126613	5.88	4.330E-06
P57780	Alpha-actinin-4	104977	5.88	4.922E-06
P24472	Glutathione S-transferase A4	25564	5.88	4.695E-05
P21550	Beta-enolase	47025	5.88	5.314E-05
Q7TPR4	Alpha-actinin-1	103068	5.88	6.143E-05
Q9JMH6	Thioredoxin reductase 1 cytoplasmic	67084	6.25	5.896E-06
Q8C1B7	Septin-11	49695	6.25	1.844E-05
Q7M6Y3	Phosphatidylinositol-binding clathrin assembly protein	71543	6.25	3.113E-05
Q99KI0	Aconitate hydratase mitochondrial	85464	6.25	3.440E-05
Q9CR60	Vesicle transport protein GOT1B	15422	6.67	1.254E-05
P16546	Spectrin alpha chain non-erythrocytic 1	284596	6.67	1.126E-04
Q9WTQ5	A-kinase anchor protein 12	180694	6.67	2.184E-03
P47791	Glutathione reductase mitochondrial	53663	7.69	3.158E-04
Q3U7R1	Extended synaptotagmin-1	121553	8.33	2.035E-06
P38647	Stress-70 protein mitochondrial	73461	8.33	3.254E-05
Q6IRU2	Tropomyosin alpha-4 chain	28468	9.09	1.076E-04
P21107	Tropomyosin alpha-3 chain	32994	11.11	3.437E-07
P19324	Serpin H1	46534	11.11	1.771E-05
P35564	Calnexin	67278	11.11	6.595E-05
P09103	Protein disulfide-isomerase	57059	12.50	3.149E-04
Q922R8	Protein disulfide-isomerase A6	48100	14.29	1.454E-04

P08113	Endoplasmin	92476	16.67	2.082E-06
P08249	Malate dehydrogenase mitochondrial	35611	16.67	1.380E-05
P14211	Calreticulin	47995	16.67	6.940E-05
P20029	Endoplasmic reticulum chaperone BiP	72422	20.00	7.941E-07
P27773	Protein disulfide-isomerase A3	56678	20.00	2.271E-05
P63038	60 kDa heat shock protein mitochondrial	60956	25.00	2.946E-06
Q9DBG6	Dolichyl-diphosphooligosaccharide--protein glycosyltransferase subunit 2	69063	25.00	2.886E-05
P56480	ATP synthase subunit beta mitochondrial	56301	33.33	1.264E-05
Q64433	10 kDa heat shock protein mitochondrial	10963	33.33	1.891E-05
Q91W90	Thioredoxin domain-containing protein 5	46416	33.33	4.098E-05

Table S2. Proteins Common to Large and Small Evs without differences in relative abundances

Accession	Description	Avg. Mass
Q8VDP3	[F-actin]-monooxygenase MICAL1	116785
Q9CQV8	14-3-3 protein beta/alpha	28086
P51432	1-phosphatidylinositol 4 5-bisphosphate phosphodiesterase beta-3	139487
Q8R3B1	1-phosphatidylinositol 4 5-bisphosphate phosphodiesterase delta-1	85873
P14685	26S proteasome non-ATPase regulatory subunit 3	60718
O35226	26S proteasome non-ATPase regulatory subunit 4	40704
Q8BJY1	26S proteasome non-ATPase regulatory subunit 5	55972
Q99JI4	26S proteasome non-ATPase regulatory subunit 6	45536
Q9CX56	26S proteasome non-ATPase regulatory subunit 8	39930
Q921H8	3-ketoacyl-CoA thiolase A peroxisomal	43953
P63323	40S ribosomal protein S12	14525
Q9CZX8	40S ribosomal protein S19	16085
Q9CQR2	40S ribosomal protein S21	9141
P62862	40S ribosomal protein S30	6648
P62867	40S ribosomal protein S30	6648
P62082	40S ribosomal protein S7	22127
Q9JM14	5'(3')-deoxyribonucleotidase cytosolic type	23076
P70290	55 kDa erythrocyte membrane protein	52227
O54950	5'-AMP-activated protein kinase subunit gamma-1	37520
P47955	60S acidic ribosomal protein P1	11475
P99027	60S acidic ribosomal protein P2	11651
P67984	60S ribosomal protein L22	14759
Q9D7S7	60S ribosomal protein L22-like 1	14467
P62751	60S ribosomal protein L23a	17695
P61255	60S ribosomal protein L26	17258
P62900	60S ribosomal protein L31	14463
Q6ZWV7	60S ribosomal protein L35	14553
P61514	60S ribosomal protein L37a	10275
Q9JJI8	60S ribosomal protein L38	8204

P51410	60S ribosomal protein L9	21881
Q9CQ60	6-phosphogluconolactonase	27254
Q8CBW3	Abl interactor 1	52288
P60710	Actin cytoplasmic 1	41737
P63260	Actin cytoplasmic 2	41793
Q9QZB7	Actin-related protein 10	46208
P61161	Actin-related protein 2	44761
Q9WV32	Actin-related protein 2/3 complex subunit 1B	41064
Q9CVB6	Actin-related protein 2/3 complex subunit 2	34357
P59999	Actin-related protein 2/3 complex subunit 4	19667
Q9CPW4	Actin-related protein 2/3 complex subunit 5	16288
Q99JY9	Actin-related protein 3	47357
Q8BK64	Activator of 90 kDa heat shock protein ATPase homolog 1	38117
P50247	Adenosylhomocysteinase	47688
P46664	Adenylosuccinate synthetase isozyme 2	50021
P61750	ADP-ribosylation factor 4	20397
P62331	ADP-ribosylation factor 6	20082
P61211	ADP-ribosylation factor-like protein 1	20412
Q9D0J4	ADP-ribosylation factor-like protein 2	20864
Q8BGQ7	Alanine--tRNA ligase cytoplasmic	106908
P28474	Alcohol dehydrogenase class-3	39548
Q571I9	Aldehyde dehydrogenase family 16 member A1	84756
P45376	Aldose reductase	35732
P45377	Aldose reductase-related protein 2	36121
P61164	Alpha-centractin	42614
P27046	Alpha-mannosidase 2	131631
Q9EPC1	Alpha-parvin	42330
Q9DB05	Alpha-soluble NSF attachment protein	33190
P31230	Aminoacyl tRNA synthase complex-interacting multifunctional protein 1	33997
Q8R010	Aminoacyl tRNA synthase complex-interacting multifunctional protein 2	35378
P97449	Aminopeptidase N	109651
P97384	Annexin A11	54079

P07356	Annexin A2	38676
P97429	Annexin A4	35916
P14824	Annexin A6	75885
Q07076	Annexin A7	49925
Q6P9J9	Anoctamin-6	106255
P17426	AP-2 complex subunit alpha-1	107664
P17427	AP-2 complex subunit alpha-2	104016
P84091	AP-2 complex subunit mu	49655
Q9Z1T1	AP-3 complex subunit beta-1	122740
Q02013	Aquaporin-1	28793
Q9WTY4	Aquaporin-5	28274
Q9D0I9	Arginine--tRNA ligase cytoplasmic	75674
Q61024	Asparagine synthetase [glutamine-hydrolyzing]	64283
Q922B2	Aspartate--tRNA ligase cytoplasmic	57147
Q9Z2W0	Aspartyl aminopeptidase	52207
P28658	Ataxin-10	53707
Q9DC29	ATP-binding cassette sub-family B member 6 mitochondrial	93770
P61222	ATP-binding cassette sub-family E member 1	67314
Q6P542	ATP-binding cassette sub-family F member 1	94945
Q91V92	ATP-citrate synthase	119728
Q91VR5	ATP-dependent RNA helicase DDX1	82500
O88738	Baculoviral IAP repeat-containing protein 6	532176
Q9JLV1	BAG family molecular chaperone regulator 3	61860
O70318	Band 4.1-like protein 2	109940
Q9WV92	Band 4.1-like protein 3	103338
Q9CQC6	Basic leucine zipper and W2 domain-containing protein 1	48043
Q8BFZ3	Beta-actin-like protein 2	42004
Q8R5C5	Beta-centractin	42281
Q60967	Bifunctional 3'-phosphoadenosine 5'-phosphosulfate synthase 1	70794
Q9CWJ9	Bifunctional purine biosynthesis protein PURH	64217
Q91XV3	Brain acid soluble protein 1	22087
Q8K2Q7	BRO1 domain-containing protein BROX	46202

Q06138	Calcium-binding protein 39	39843
O08529	Calpain-2 catalytic subunit	79872
Q9DAW9	Calponin-3	36429
Q9DBC7	cAMP-dependent protein kinase type I-alpha regulatory subunit	43185
P08074	Carbonyl reductase [NADPH] 2	25958
O89001	Carboxypeptidase D	152405
P26231	Catenin alpha-1	100106
Q61301	Catenin alpha-2	105286
Q02248	Catenin beta-1	85471
P10605	Cathepsin B	37280
P24668	Cation-dependent mannose-6-phosphate receptor	31172
O35566	CD151 antigen	28246
Q8VE98	CD276 antigen	34002
P15379	CD44 antigen	85617
P41731	CD63 antigen	25767
P40240	CD9 antigen	25258
Q9Z0M6	CD97 antigen	90413
Q8VCN6	CD99 antigen	16783
P60766	Cell division control protein 42 homolog	21259
Q08288	Cell growth-regulating nucleolar protein	43736
Q5FWI3	Cell surface hyaluronidase	153800
Q9D8B3	Charged multivesicular body protein 4b	24936
Q9D7S9	Charged multivesicular body protein 5	24576
Q9Z1Q5	Chloride intracellular channel protein 1	27013
Q6X893	Choline transporter-like protein 1	73067
P23198	Chromobox protein homolog 3	20855
Q58A65	C-Jun-amino-terminal kinase-interacting protein 4	146219
Q68FD5	Clathrin heavy chain 1	191555
O08585	Clathrin light chain A	25604
O55029	Coatomer subunit beta'	102449
Q5XJY5	Coatomer subunit delta	57230
O89079	Coatomer subunit epsilon	34567

Q9QZE5	Coatomer subunit gamma-1	97513
Q9JIG7	Coiled-coil domain-containing protein 22	70844
Q91W50	Cold shock domain-containing protein E1	88791
P11087	Collagen alpha-1(I) chain	138033
P08121	Collagen alpha-1(III) chain	138944
Q01149	Collagen alpha-2(I) chain	129557
Q64735	Complement component receptor 1-like protein	53763
Q8K2Z4	Condensin complex subunit 1	155664
P61202	COP9 signalosome complex subunit 2	51597
Q9CZ04	COP9 signalosome complex subunit 7a	30224
Q8C166	Copine-1	58887
Q8BT60	Copine-3	59585
Q04447	Creatine kinase B-type	42713
P70698	CTP synthase 1	66683
Q9WTX6	Cullin-1	89691
Q9D1P4	Cysteine and histidine-rich domain-containing protein 1	37351
O88487	Cytoplasmic dynein 1 intermediate chain 2	68394
Q8R1Q8	Cytoplasmic dynein 1 light intermediate chain 1	56614
Q6PDL0	Cytoplasmic dynein 1 light intermediate chain 2	54219
Q7TMB8	Cytoplasmic FMR1-interacting protein 1	145241
Q9CPY7	Cytosol aminopeptidase	56141
Q91V12	Cytosolic acyl coenzyme A thioester hydrolase	42537
P47713	Cytosolic phospholipase A2	85222
Q61753	D-3-phosphoglycerate dehydrogenase	56586
O35215	D-dopachrome decarboxylase	13077
Q8CJ26	Death domain-containing membrane protein NRADD	24727
Q8R1A4	Dedicator of cytokinesis protein 7	241436
Q91YP3	Deoxyribose-phosphate aldolase	34975
P31001	Desmin	53498
P32233	Developmentally-regulated GTP-binding protein 1	40512
O08553	Dihydropyrimidinase-related protein 2	62278
Q8BWT5	Disco-interacting protein 2 homolog A	165254

Q61072	Disintegrin and metalloproteinase domain-containing protein 9	92080
Q811D0	Disks large homolog 1	100120
P13864	DNA (cytosine-5)-methyltransferase 1	183188
P97310	DNA replication licensing factor MCM2	102077
Q01320	DNA topoisomerase 2-alpha	172789
P28352	DNA-(apurinic or apyrimidinic site) lyase	35490
P60904	DnaJ homolog subfamily C member 5	22101
Q91YQ5	Dolichyl-diphosphooligosaccharide--protein glycosyltransferase subunit 1	68528
Q9Z108	Double-stranded RNA-binding protein Staufen homolog 1	53925
Q62418	Drebrin-like protein	48700
O08788	Dynactin subunit 1	141675
Q99KJ8	Dynactin subunit 2	44117
Q9Z0Y1	Dynactin subunit 3	20978
Q8CBY8	Dynactin subunit 4	53057
Q8K1M6	Dynamin-1-like protein	82658
P39054	Dynamin-2	98145
Q9ERU9	E3 SUMO-protein ligase RanBP2	341122
Q7TMY8	E3 ubiquitin-protein ligase HUWE1	482640
P46935	E3 ubiquitin-protein ligase NEDD4	102706
A2AN08	E3 ubiquitin-protein ligase UBR4	572298
P06802	Ectonucleotide pyrophosphatase/phosphodiesterase family member 1	103176
O35474	EGF-like repeat and discoidin I-like domain-containing protein 3	53712
E9PVA8	eIF-2-alpha kinase activator GCN1	293018
P10126	Elongation factor 1-alpha 1	50114
P57776	Elongation factor 1-delta	31293
Q9D8N0	Elongation factor 1-gamma	50061
P58252	Elongation factor 2	95314
Q62419	Endophilin-A2	41518
Q9JK48	Endophilin-B1	40855
Q4PZA2	Endothelin-converting enzyme 1	87073
P00924	Enolase 1	46816
Q03145	Ephrin type-A receptor 2	108852

Q9JIM1	Equilibrative nucleoside transporter 1	50192
Q80TH2	Erbin	157248
P54116	Erythrocyte band 7 integral membrane protein	31375
Q8R050	Eukaryotic peptide chain release factor GTP-binding subunit ERF3A	68626
Q6ZWX6	Eukaryotic translation initiation factor 2 subunit 1	36108
Q99L45	Eukaryotic translation initiation factor 2 subunit 2	38092
Q9Z0N1	Eukaryotic translation initiation factor 2 subunit 3 X-linked	51066
Q8BJW6	Eukaryotic translation initiation factor 2A	64403
P23116	Eukaryotic translation initiation factor 3 subunit A	161936
Q8JZQ9	Eukaryotic translation initiation factor 3 subunit B	91370
Q8R1B4	Eukaryotic translation initiation factor 3 subunit C	105531
P60229	Eukaryotic translation initiation factor 3 subunit E	52221
Q9DCH4	Eukaryotic translation initiation factor 3 subunit F	37984
Q9Z1D1	Eukaryotic translation initiation factor 3 subunit G	35638
Q91WK2	Eukaryotic translation initiation factor 3 subunit H	39832
Q9QZD9	Eukaryotic translation initiation factor 3 subunit I	36461
Q8QZY1	Eukaryotic translation initiation factor 3 subunit L	66613
Q99JX4	Eukaryotic translation initiation factor 3 subunit M	42517
Q6NZJ6	Eukaryotic translation initiation factor 4 gamma 1	176076
Q8BGD9	Eukaryotic translation initiation factor 4B	68840
P63073	Eukaryotic translation initiation factor 4E	25053
P59325	Eukaryotic translation initiation factor 5	48968
Q05D44	Eukaryotic translation initiation factor 5B	137616
O55135	Eukaryotic translation initiation factor 6	26511
A6H5Z3	Exocyst complex component 6B	94130
Q6P5F9	Exportin-1	123093
Q9ERK4	Exportin-2	110455
Q3TZZ7	Extended synaptotagmin-2	94139
P47753	F-actin-capping protein subunit alpha-1	32940
P47754	F-actin-capping protein subunit alpha-2	32967
P47757	F-actin-capping protein subunit beta	31345
Q3U0V1	Far upstream element-binding protein 2	76776

P19096	Fatty acid synthase	272426
B2RXV4	Feline leukemia virus subgroup C receptor-related protein 1	60469
F8VPU2	FERM ARHGEF and pleckstrin domain-containing protein 1	118875
Q8CIB5	Fermitin family homolog 2	77800
P09528	Ferritin heavy chain	21067
P29391	Ferritin light chain 1	20802
Q8VHX6	Filamin-C	291117
Q923D2	Flavin reductase (NADPH)	22197
O08917	Flotillin-1	47513
Q6ZPF4	Formin-like protein 3	117169
Q9R059	Four and a half LIM domains protein 3	31795
Q61584	Fragile X mental retardation syndrome-related protein 1	76222
P97807	Fumarate hydratase mitochondrial	54357
P16110	Galectin-3	27515
Q8K0C9	GDP-mannose 4 6 dehydratase	41985
P13020	Gelsolin	85942
Q00612	Glucose-6-phosphate 1-dehydrogenase X	59263
O09172	Glutamate--cysteine ligase regulatory subunit	30535
P47856	Glutamine--fructose-6-phosphate aminotransferase [isomerizing] 1	78539
Q8BML9	Glutamine--tRNA ligase	87677
P10649	Glutathione S-transferase Mu 1	25970
P19157	Glutathione S-transferase P 1	23609
P16858	Glyceraldehyde-3-phosphate dehydrogenase	35810
Q9CZD3	Glycine--tRNA ligase	81878
Q9JHJ3	Glycosylated lysosomal membrane protein	43804
O70310	Glycylpeptide N-tetradecanoyltransferase 1	56888
Q9QZF2	Glypican-1	61360
Q3THK7	GMP synthase [glutamine-hydrolyzing]	76724
Q61543	Golgi apparatus protein 1	133734
Q99JX3	Golgi reassembly-stacking protein 2	47038
Q8BMP6	Golgi resident protein GCP60	60181
Q9CYL5	Golgi-associated plant pathogenesis-related protein 1	17090

P08556	GTPase NRas	21199
O08582	GTP-binding protein 1	72301
P36536	GTP-binding protein SAR1a	22371
P08752	Guanine nucleotide-binding protein G(i) subunit alpha-2	40489
Q9DC51	Guanine nucleotide-binding protein G(k) subunit alpha	40538
P18872	Guanine nucleotide-binding protein G(o) subunit alpha	40085
P21279	Guanine nucleotide-binding protein G(q) subunit alpha	42158
P63094	Guanine nucleotide-binding protein G(s) subunit alpha isoforms short	45664
P27601	Guanine nucleotide-binding protein subunit alpha-13	44055
P29387	Guanine nucleotide-binding protein subunit beta-4	37379
Q9WVD4	H(+)/Cl(-) exchange transporter 5	83101
P17879	Heat shock 70 kDa protein 1B	70176
P16627	Heat shock 70 kDa protein 1-like	70637
P63017	Heat shock cognate 71 kDa protein	70871
Q61699	Heat shock protein 105 kDa	96407
P14602	Heat shock protein beta-1	23014
Q99LI8	Hepatocyte growth factor-regulated tyrosine kinase substrate	86015
Q8BG05	Heterogeneous nuclear ribonucleoprotein A3	39652
Q9Z2X1	Heterogeneous nuclear ribonucleoprotein F	45730
P61979	Heterogeneous nuclear ribonucleoprotein K	50976
Q9D0E1	Heterogeneous nuclear ribonucleoprotein M	77649
Q7TMK9	Heterogeneous nuclear ribonucleoprotein Q	69633
O88569	Heterogeneous nuclear ribonucleoproteins A2/B1	37403
P62748	Hippocalcin-like protein 1	22338
P43275	Histone H1.1	21785
P15864	Histone H1.2	21267
Q60973	Histone-binding protein RBBP7	47790
Q8JZK9	Hydroxymethylglutaryl-CoA synthase cytoplasmic	57569
P00493	Hypoxanthine-guanine phosphoribosyltransferase	24570
Q6ZQA6	Immunoglobulin superfamily member 3	134710
P52293	Importin subunit alpha-1	57928
O35345	Importin subunit alpha-7	59964

P70168	Importin subunit beta-1	97184
P24547	Inosine-5'-monophosphate dehydrogenase 2	55815
Q80V26	Inositol monophosphatase 3	38616
P11688	Integrin alpha-5	115043
Q61739	Integrin alpha-6	122159
Q61738	Integrin alpha-7	129329
P09055	Integrin beta-1	88231
Q99J93	Interferon-induced transmembrane protein 2	15743
Q9CXY6	Interleukin enhancer-binding factor 2	43062
Q0GNC1	Inverted formin-2	138560
Q9CX00	IST1 homolog	39468
Q02257	Junction plakoglobin	81801
P02535	Keratin type I cytoskeletal 10	57770
Q9Z2K1	Keratin type I cytoskeletal 16	51606
Q6IFX2	Keratin type I cytoskeletal 42	50133
P04104	Keratin type II cytoskeletal 1	65606
Q3TTY5	Keratin type II cytoskeletal 2 epidermal	70923
Q922U2	Keratin type II cytoskeletal 5	61767
Q80W68	Kin of IRRE-like protein 1	87176
O88447	Kinesin light chain 1	61450
Q61768	Kinesin-1 heavy chain	109551
P21956	Lactadherin	51241
P02469	Laminin subunit beta-1	197089
Q9Z1R2	Large proline-rich protein BAG6	121037
Q505F5	Leucine-rich repeat-containing protein 47	63590
Q922Q8	Leucine-rich repeat-containing protein 59	34877
P24527	Leukotriene A-4 hydrolase	69051
Q99JW4	LIM and senescent cell antigen-like-containing domain protein 1	37240
Q8BFW7	Lipoma-preferred partner homolog	65891
Q9QUJ7	Long-chain-fatty-acid--CoA ligase 4	79077
Q9D358	Low molecular weight phosphotyrosine protein phosphatase	18192
Q64253	Lymphocyte antigen 6E	13800

O35114	Lysosome membrane protein 2	54044
P11438	Lysosome-associated membrane glycoprotein 1	43865
P14152	Malate dehydrogenase cytoplasmic	36511
Q8CI59	Metalloreductase STEAP3	54749
O08663	Methionine aminopeptidase 2	52922
P27546	Microtubule-associated protein 4	117429
Q61166	Microtubule-associated protein RP/EB family member 1	30016
Q9CYG7	Mitochondrial import receptor subunit TOM34	34278
P63085	Mitogen-activated protein kinase 1	41276
P97820	Mitogen-activated protein kinase kinase kinase kinase 4	140602
P10404	MLV-related proviral Env polyprotein	69613
P06795	Multidrug resistance protein 1B	140993
Q9DCL9	Multifunctional protein ADE2	47006
Q8VCR8	Myosin light chain kinase 2 skeletal/cardiac muscle	65990
Q60605	Myosin light polypeptide 6	16930
Q3THE2	Myosin regulatory light chain 12B	19779
Q8VDD5	Myosin-9	226370
Q99LD8	N(G) N(G)-dimethylarginine dimethylaminohydrolase 2	29646
Q64669	NAD(P)H dehydrogenase [quinone] 1	30960
Q80UM3	N-alpha-acetyltransferase 15 NatA auxiliary subunit	100961
P28660	Nck-associated protein 1	128784
O35516	Neurogenic locus notch homolog protein 2	265326
P05480	Neuronal proto-oncogene tyrosine-protein kinase Src	60645
P97333	Neuropilin-1	103000
O35874	Neutral amino acid transporter A	56062
P51912	Neutral amino acid transporter B(0)	58483
P10493	Nidogen-1	136538
Q99K48	Non-POU domain-containing octamer-binding protein	54541
O35604	NPC intracellular cholesterol transporter 1	142884
Q9Z0J0	NPC intracellular cholesterol transporter 2	16442
Q9CZ44	NSFL1 cofactor p47	40710
Q99MD9	Nuclear autoantigenic sperm protein	83954

Q3UYV9	Nuclear cap-binding protein subunit 1	91927
P62960	Nuclease-sensitive element-binding protein 1	35730
Q6DFW4	Nucleolar protein 58	60343
Q9JIK5	Nucleolar RNA helicase 2	93551
P09405	Nucleolin	76723
Q61937	Nucleophosmin	32560
P28656	Nucleosome assembly protein 1-like 1	45345
Q9CZ30	Obg-like ATPase 1	44730
Q3B7Z2	Oxysterol-binding protein 1	88797
Q9Z0P4	Paralemmin-1	41614
Q8CI51	PDZ and LIM domain protein 5	63299
Q3TJD7	PDZ and LIM domain protein 7	50119
Q8BFY6	Peflin	29228
P30412	Peptidyl-prolyl cis-trans isomerase C	22794
Q9DBG5	Perilipin-3	47262
O70589	Peripheral plasma membrane protein CASK	105109
P51660	Peroxisomal multifunctional enzyme type 2	79482
Q8C0C7	Phenylalanine--tRNA ligase alpha subunit	57599
Q9WUA2	Phenylalanine--tRNA ligase beta subunit	65697
P70296	Phosphatidylethanolamine-binding protein 1	20830
E9Q3L2	Phosphatidylinositol 4-kinase alpha	237040
P09411	Phosphoglycerate kinase 1	44550
Q9D0M1	Phosphoribosyl pyrophosphate synthase-associated protein 1	39432
Q5SUR0	Phosphoribosylformylglycinamidine synthase	144629
P63005	Platelet-activating factor acetylhydrolase IB subunit alpha	46670
P05622	Platelet-derived growth factor receptor beta	122806
Q9QXS1	Plectin	534193
P70206	Plexin-A1	211098
B2RXS4	Plexin-B2	206229
P60335	Poly(rC)-binding protein 1	37498
Q61990	Poly(rC)-binding protein 2	38222
Q3UEB3	Poly(U)-binding-splicing factor PUF60	60249

P29341	Polyadenylate-binding protein 1	70671
P17225	Polypyrimidine tract-binding protein 1	56478
O70591	Prefoldin subunit 2	16534
Q99KP6	Pre-mRNA-processing factor 19	55239
P49769	Presenilin-1	52640
Q922K7	Probable 28S rRNA (cytosine-C(5))-methyltransferase	86752
P54823	Probable ATP-dependent RNA helicase DDX6	54192
P70398	Probable ubiquitin carboxyl-terminal hydrolase FAF-X	290709
Q8K297	Procollagen galactosyltransferase 1	71061
Q9JHZ2	Progressive ankylosis protein	54298
P17918	Proliferating cell nuclear antigen	28785
P50580	Proliferation-associated protein 2G4	43699
Q91ZX7	Prolow-density lipoprotein receptor-related protein 1	504745
Q60715	Prolyl 4-hydroxylase subunit alpha-1	60910
Q9QUR6	Prolyl endopeptidase	80752
Q61207	Prosaposin	61422
Q91YR9	Prostaglandin reductase 1	35560
P61290	Proteasome activator complex subunit 3	29506
Q9R1P4	Proteasome subunit alpha type-1	29547
O70435	Proteasome subunit alpha type-3	28405
Q9R1P0	Proteasome subunit alpha type-4	29471
Q9Z2U1	Proteasome subunit alpha type-5	26411
Q9QUM9	Proteasome subunit alpha type-6	27372
Q9Z2U0	Proteasome subunit alpha type-7	27855
O09061	Proteasome subunit beta type-1	26372
Q9R1P3	Proteasome subunit beta type-2	22906
Q9R1P1	Proteasome subunit beta type-3	22965
P99026	Proteasome subunit beta type-4	29116
Q60692	Proteasome subunit beta type-6	25379
P70195	Proteasome subunit beta type-7	29891
Q9JIF0	Protein arginine N-methyltransferase 1	42436
Q8CIG8	Protein arginine N-methyltransferase 5	72680

Q8BXZ1	Protein disulfide-isomerase TMX3	51848
Q8C0Z1	Protein FAM234A	60576
Q9JJ28	Protein flightless-1 homolog	144803
Q99JB8	Protein kinase C and casein kinase II substrate protein 3	48585
Q9WVE8	Protein kinase C and casein kinase substrate in neurons protein 2	55833
Q3UM45	Protein phosphatase 1 regulatory subunit 7	41292
Q8BK67	Protein RCC2	55983
P08207	Protein S100-A10	11186
P07091	Protein S100-A4	11721
Q80U72	Protein scribble homolog	174058
Q01405	Protein transport protein Sec23A	86162
Q3UPL0	Protein transport protein Sec31A	133569
Q80WQ2	Protein VAC14 homolog	88048
P23492	Purine nucleoside phosphorylase	32277
P23249	Putative helicase MOV-10	113583
Q810B6	Rabankyrin-5	128652
Q9CQ22	Ragulator complex protein LAMTOR1	17749
P34022	Ran-specific GTPase-activating protein	23596
Q01730	Ras suppressor protein 1	31550
P63001	Ras-related C3 botulinum toxin substrate 1	21450
Q99K70	Ras-related GTP-binding protein C	44121
P61027	Ras-related protein Rab-10	22541
Q91V41	Ras-related protein Rab-14	23897
P35293	Ras-related protein Rab-18	23035
P62821	Ras-related protein Rab-1A	22678
Q9D1G1	Ras-related protein Rab-1B	22187
P35282	Ras-related protein Rab-21	24106
Q6PHN9	Ras-related protein Rab-35	23025
Q9CQD1	Ras-related protein Rab-5A	23599
P35278	Ras-related protein Rab-5C	23412
P35279	Ras-related protein Rab-6A	23590
P51150	Ras-related protein Rab-7a	23490

Q9JIW9	Ras-related protein Ral-B	23349
P62835	Ras-related protein Rap-1A	20987
Q99JI6	Ras-related protein Rap-1b	20825
P10833	Ras-related protein R-Ras	23764
P62071	Ras-related protein R-Ras2	23400
Q8K2J7	RELT-like protein 1	29351
Q8BHL4	Retinoic acid-induced protein 3	40101
Q60875	Rho guanine nucleotide exchange factor 2	111974
P70336	Rho-associated protein kinase 2	160585
P62746	Rho-related GTP-binding protein RhoB	22123
Q62159	Rho-related GTP-binding protein RhoC	22006
P84096	Rho-related GTP-binding protein RhoG	21308
Q9D7G0	Ribose-phosphate pyrophosphokinase 1	34834
Q99PL5	Ribosome-binding protein 1	172878
Q8VH51	RNA-binding protein 39	59407
O35609	Secretory carrier-associated membrane protein 3	38458
Q64105	Sepiapterin reductase	27883
O55131	Septin-7	50550
Q64337	Sequestosome-1	48163
Q6PDM2	Serine/arginine-rich splicing factor 1	27745
P84104	Serine/arginine-rich splicing factor 3	19330
Q8BL97	Serine/arginine-rich splicing factor 7	30818
Q8CIN4	Serine/threonine-protein kinase PAK 2	57930
Q6P1F6	Serine/threonine-protein phosphatase 2A 55 kDa regulatory subunit B alpha isoform	51692
Q76MZ3	Serine/threonine-protein phosphatase 2A 65 kDa regulatory subunit A alpha isoform	65323
P63330	Serine/threonine-protein phosphatase 2A catalytic subunit alpha isoform	35608
P26638	Serine--tRNA ligase cytoplasmic	58389
Q99J77	Sialic acid synthase	40024
Q8BMA6	Signal recognition particle subunit SRP68	70574
P70297	Signal transducing adapter molecule 1	59771
Q91Z67	SLIT-ROBO Rho GTPase-activating protein 2	120798
P62317	Small nuclear ribonucleoprotein Sm D2	13527

P62320	Small nuclear ribonucleoprotein Sm D3	13916
Q9CQ65	S-methyl-5'-thioadenosine phosphorylase	31062
O35316	Sodium- and chloride-dependent taurine transporter	69856
Q8BTY2	Sodium bicarbonate cotransporter 3	116514
Q9JIS8	Solute carrier family 12 member 4	120624
Q9WVL3	Solute carrier family 12 member 7	119481
Q6P069	Sorcin	21627
Q9WV80	Sorting nexin-1	58952
Q91ZR2	Sorting nexin-18	67904
Q6P8X1	Sorting nexin-6	46649
Q91VH2	Sorting nexin-9	66546
Q64674	Spermidine synthase	33995
Q9WTX5	S-phase kinase-associated protein 1	18672
Q8VIJ6	Splicing factor proline- and glutamine-rich	75442
Q9D554	Splicing factor 3A subunit 3	58842
Q78PY7	Staphylococcal nuclease domain-containing protein 1	102088
O54988	STE20-like serine/threonine-protein kinase	141457
Q9WUD1	STIP1 homology and U box-containing protein 1	34909
Q9R1T2	SUMO-activating enzyme subunit 1	38620
Q9Z1F9	SUMO-activating enzyme subunit 2	70569
P35922	Synaptic functional regulator FMR1	68989
O09044	Synaptosomal-associated protein 23	23261
Q9ER00	Syntaxin-12	31195
P70452	Syntaxin-4	34165
O88983	Syntaxin-8	26925
O08599	Syntaxin-binding protein 1	67569
Q60770	Syntaxin-binding protein 3	67943
P26039	Talin-1	269819
Q71LX4	Talin-2	253618
P47226	Testin	47983
O70401	Tetraspanin-6	27333
Q8CDN6	Thioredoxin-like protein 1	32237

Q9D0R2	Threonine--tRNA ligase cytoplasmic	83356
Q00609	T-lymphocyte activation antigen CD80	34590
Q93092	Transaldolase	37387
Q62318	Transcription intermediary factor 1-beta	88847
P42669	Transcriptional activator protein Pur-alpha	34884
O35295	Transcriptional activator protein Pur-beta	33901
Q9QUI0	Transforming protein RhoA	21782
Q01853	Transitional endoplasmic reticulum ATPase	89322
P40142	Transketolase	67631
P58021	Transmembrane 9 superfamily member 2	75330
Q9ET30	Transmembrane 9 superfamily member 3	67545
Q80X71	Transmembrane protein 106B	31172
Q8BFY9	Transportin-1	102357
Q64737	Trifunctional purine biosynthetic protein adenosine-3	107503
P68369	Tubulin alpha-1A chain	50136
Q922F4	Tubulin beta-6 chain	50090
P83887	Tubulin gamma-1 chain	51101
Q8BYA0	Tubulin-specific chaperone D	133321
Q6PB44	Tyrosine-protein phosphatase non-receptor type 23	185215
Q62376	U1 small nuclear ribonucleoprotein 70 kDa	51992
Q62189	U1 small nuclear ribonucleoprotein A	31835
Q9JMA1	Ubiquitin carboxyl-terminal hydrolase 14	56002
Q7TQI3	Ubiquitin thioesterase OTUB1	31270
P62838	Ubiquitin-conjugating enzyme E2 D2	16735
P61079	Ubiquitin-conjugating enzyme E2 D3	16687
P61089	Ubiquitin-conjugating enzyme E2 N	17138
Q9CZY3	Ubiquitin-conjugating enzyme E2 variant 1	16355
Q02053	Ubiquitin-like modifier-activating enzyme 1	117809
Q8C7R4	Ubiquitin-like modifier-activating enzyme 6	117966
O70475	UDP-glucose 6-dehydrogenase	54832
Q3TW96	UDP-N-acetylhexosamine pyrophosphorylase-like protein 1	56614
Q9DBP5	UMP-CMP kinase	22165

Q9WTI7	Unconventional myosin-Ic	121944
Q9JK81	UPF0160 protein MYG1 mitochondrial	42723
P13439	Uridine 5'-monophosphate synthase	52292
P35456	Urokinase plasminogen activator surface receptor	35428
P40336	Vacuolar protein sorting-associated protein 26A	38114
Q9EQH3	Vacuolar protein sorting-associated protein 35	91713
Q9CR26	Vacuolar protein sorting-associated protein VTA1 homolog	33913
P70460	Vasodilator-stimulated phosphoprotein	39667
O88384	Vesicle transport through interaction with t-SNAREs homolog 1B	26713
Q8VDJ3	Vigilin	141742
P20152	Vimentin	53688
O08532	Voltage-dependent calcium channel subunit alpha-2/delta-1	124630
P50516	V-type proton ATPase catalytic subunit A	68326
P62814	V-type proton ATPase subunit B brain isoform	56551
P57746	V-type proton ATPase subunit D	28369
P51863	V-type proton ATPase subunit d 1	40301
P50518	V-type proton ATPase subunit E 1	26157
Q8BVE3	V-type proton ATPase subunit H	55855
Q6PGL7	WASH complex subunit 2	145310
Q3UMB9	WASH complex subunit 4	136370
Q8C2E7	WASH complex subunit 5	134110
Q9ERF3	WD repeat-containing protein 61	33773
Q8BH43	Wiskott-Aldrich syndrome protein family member 2	54074
Q6P1B1	Xaa-Pro aminopeptidase 1	69591
Q9JKB3	Y-box-binding protein 3	38814
Q7TMA2	Zinc finger protein 503	63036

Table S3. Proteins exclusively present in Large Evs. For protein Acc. No, description, -10lgP, sequence coverage, the number of identified peptides and the number of unique peptides are reported.

Accession	Description	Avg. Mass	-10lgP	Coverage (%)	Peptides	Unique peptides
P58871	182 kDa tankyrase-1-binding protein	181824	124.47	3	3	3
P16330	2' 3'-cyclic-nucleotide 3'-phosphodiesterase	47123	81.32	10	2	2
Q3UHX2	28 kDa heat- and acid-stable phosphoprotein	20605	99.21	10	2	2
Q80VJ3	2'-deoxynucleoside 5'-phosphate N-hydrolase 1	18977	53.37	18	2	2
Q60597	2-oxoglutarate dehydrogenase mitochondrial	116449	90.99	7	5	5
Q99N96	39S ribosomal protein L1 mitochondrial	37597	38.78	9	2	2
Q9DB15	39S ribosomal protein L12 mitochondrial	21708	108.95	31	5	5
P70245	3-beta-hydroxysteroid-Delta(8) Delta(7)-isomerase	26215	97.23	10	2	2
Q8BWT1	3-ketoacyl-CoA thiolase mitochondrial	41830	146	16	4	4
P53026	60S ribosomal protein L10a	24916	157.58	31	7	7
Q8BU33	Acetolactate synthase-like protein	68156	141.14	10	3	3
Q8QZT1	Acetyl-CoA acetyltransferase mitochondrial	44816	147.75	31	7	7
Q99J27	Acetyl-coenzyme A transporter 1	61075	78.43	7	2	2
Q9WV54	Acid ceramidase	44670	61.96	8	3	3
O35381	Acidic leucine-rich nuclear phosphoprotein 32 family member A	28538	133.3	23	3	3
Q9EST5	Acidic leucine-rich nuclear phosphoprotein 32 family member B	31079	68.01	11	2	2
P97822	Acidic leucine-rich nuclear phosphoprotein 32 family member E	29622	123.95	19	3	3
Q8JZN5	Acyl-CoA dehydrogenase family member 9 mitochondrial	68722	69.45	8	2	2
Q9R0X4	Acyl-coenzyme A thioesterase 9 mitochondrial	50561	149.69	17	6	6
P97823	Acyl-protein thioesterase 1	24688	106.76	21	3	3
Q64010	Adapter molecule crk	33815	88.6	16	3	3
Q9WTP6	Adenylate kinase 2 mitochondrial	26469	172.92	31	6	5
Q9R0Y5	Adenylate kinase isoenzyme 1	21540	69.37	13	2	2
P54822	Adenylosuccinate lyase	54866	167.01	35	10	10
P48962	ADP/ATP translocase 1	32904	250.79	57	17	7
P51881	ADP/ATP translocase 2	32931	244.85	48	16	6
Q8VDL4	ADP-dependent glucokinase	53903	132.32	19	6	6
Q9JKX6	ADP-sugar pyrophosphatase	23984	50.25	13	2	2

O08915	AH receptor-interacting protein	37605	38.81	9	2	2
P47738	Aldehyde dehydrogenase mitochondrial	56538	194.65	31	11	10
Q9JII6	Aldo-keto reductase family 1 member A1	36587	156.34	24	6	6
Q8C0I1	Alkyldihydroxyacetonephosphate synthase peroxisomal	71684	88.53	5	2	2
P55302	Alpha-2-macroglobulin receptor-associated protein	42215	123.91	13	4	4
Q9QYC0	Alpha-adducin	80647	81.58	4	2	2
Q6PAM1	Alpha-taxilin	62369	85.58	6	2	2
Q9JJW6	Aly/REF export factor 2	23730	96.8	17	2	2
Q64133	Amine oxidase [flavin-containing] A	59602	129.81	24	8	8
P12023	Amyloid-beta A4 protein	86722	47.05	3	2	2
Q8WTY4	Anamorsin	33429	78.78	18	3	3
Q9EP71	Ankycorbin	108852	47.78	4	2	2
Q8BH79	Anoctamin-10	76188	42.96	5	2	2
O35643	AP-1 complex subunit beta-1	103935	226.48	24	14	7
P22892	AP-1 complex subunit gamma-1	91350	83.52	4	3	3
P35585	AP-1 complex subunit mu-1	48543	89.84	9	2	2
P62743	AP-2 complex subunit sigma	17018	64.87	20	3	3
O54774	AP-3 complex subunit delta-1	135081	170.91	10	7	7
O35841	Apoptosis inhibitor 5	56785	183.29	20	5	5
Q8K2K6	Arf-GAP domain and FG repeat-containing protein 1	58043	39.43	5	2	2
Q91YI0	Argininosuccinate lyase	51739	115.37	16	3	3
P05201	Aspartate aminotransferase cytoplasmic	46248	233.36	40	13	13
P05202	Aspartate aminotransferase mitochondrial	47411	226.41	43	14	14
Q8BSY0	Aspartyl/asparaginyl beta-hydroxylase	83042	68.42	7	3	3
Q62048	Astrocytic phosphoprotein PEA-15	15054	69.37	30	3	3
Q80Z10	Astrotactin-2	149390	35.17	3	2	2
Q6PA06	Atlastin-2	66224	56.71	6	2	2
Q91YH5	Atlastin-3	60575	180.96	30	10	10
Q9CQQ7	ATP synthase F(0) complex subunit B1 mitochondrial	28949	78.39	23	4	4
Q03265	ATP synthase subunit alpha mitochondrial	59753	315.35	49	23	23
Q9DCX2	ATP synthase subunit d mitochondrial	18749	153.7	48	5	5
Q9D3D9	ATP synthase subunit delta mitochondrial	17600	137.39	29	3	3

Q91VR2	ATP synthase subunit gamma mitochondrial	32886	142.36	35	7	7
Q9DB20	ATP synthase subunit O mitochondrial	23364	149.02	32	5	5
P97450	ATP synthase-coupling factor 6 mitochondrial	12496	62.62	34	2	2
O54984	ATPase Asna1	38823	72.65	9	3	3
Q9Z2H5	Band 4.1-like protein 1	98315	98.85	10	6	4
Q61334	B-cell receptor-associated protein 29	27964	54.17	10	2	2
P15535	Beta-1 4-galactosyltransferase 1	44411	47.75	6	2	2
Q8BTJ4	Bis(5'-adenosyl)-triphosphatase enpp4	51611	71.29	5	2	2
Q8R016	Bleomycin hydrolase	52511	106.77	20	5	5
Q8BKX1	Brain-specific angiogenesis inhibitor 1-associated protein 2	59237	134.23	14	6	6
P24288	Branched-chain-amino-acid aminotransferase cytosolic	42791	131.06	16	5	5
Q8VC57	BTB/POZ domain-containing protein KCTD5	26164	89.58	16	2	2
Q80W54	CAAX prenyl protease 1 homolog	54735	115.93	21	6	6
Q9WTR5	Cadherin-13	78186	103.15	12	4	4
P61022	Calcineurin B homologous protein 1	22432	90.46	23	4	4
Q921L3	Calcium load-activated calcium channel	21175	102.54	12	2	2
Q8BH59	Calcium-binding mitochondrial carrier protein Aralar1	74570	158.32	14	5	5
Q8BMD8	Calcium-binding mitochondrial carrier protein SCaMC-1	52902	130.18	11	3	3
O35350	Calpain-1 catalytic subunit	82106	96.33	11	5	5
P51125	Calpastatin	84922	226.88	31	12	12
O35887	Calumenin	37064	238.51	56	12	12
P12367	cAMP-dependent protein kinase type II-alpha regulatory subunit	45389	89.38	12	3	2
P31324	cAMP-dependent protein kinase type II-beta regulatory subunit	46167	93.18	17	5	4
Q60865	Caprin-1	78169	180.17	19	8	8
Q8K354	Carbonyl reductase [NADPH] 3	30953	115.47	24	4	4
Q60737	Casein kinase II subunit alpha	45134	84.48	11	3	3
P67871	Casein kinase II subunit beta	24942	84.08	19	3	3
P70677	Caspase-3	31475	79.08	18	3	3
P24270	Catalase	59795	188.23	23	8	8
O88587	Catechol O-methyltransferase	29486	155.12	30	4	4
P06797	Cathepsin L1	37547	77.63	10	2	2
Q07113	Cation-independent mannose-6-phosphate receptor	273814	101.04	2	3	3

Q63918	Caveolae-associated protein 2	46764	103.08	7	2	2
Q91VJ2	Caveolae-associated protein 3	27853	59.97	17	2	2
Q8CJ53	Cdc42-interacting protein 4	68489	99.49	12	4	4
Q9CQB5	CDGSH iron-sulfur domain-containing protein 2	15242	89.72	21	2	2
Q9D9K3	Cell death regulator Aven	37196	47.91	9	2	2
P53996	Cellular nucleic acid-binding protein	19592	94.28	17	2	2
Q924Z4	Ceramide synthase 2	45024	105.92	11	3	3
Q9CQX5	Claudin domain-containing protein 1	28572	73.7	15	3	3
Q9CQF3	Cleavage and polyadenylation specificity factor subunit 5	26240	130.39	27	3	3
Q8VBZ3	Cleft lip and palate transmembrane protein 1 homolog	75291	163.94	20	6	6
Q80TV8	CLIP-associating protein 1	169227	94.07	5	5	5
Q9JIF7	Coatomer subunit beta	107066	225.66	23	14	14
Q9QXK3	Coatomer subunit gamma-2	97680	151.11	7	5	2
P61924	Coatomer subunit zeta-1	20198	104.36	41	4	4
Q8BRN9	Coiled-coil and C2 domain-containing protein 1B	93091	59.43	5	2	2
Q9D024	Coiled-coil domain-containing protein 47	55844	153.28	16	5	5
Q61245	Collagen alpha-1(XI) chain	181031	39.47	2	2	2
Q99MQ5	Collagen alpha-1(XXV) chain	65377	34.5	4	2	2
O35658	Complement component 1 Q subcomponent-binding protein mitochondrial	31013	169.76	54	7	7
Q99LD4	COP9 signalosome complex subunit 1	53442	69.87	9	2	2
O35864	COP9 signalosome complex subunit 5	37549	76.6	20	5	5
O08997	Copper transport protein ATOX1	7338	60.68	38	2	2
Q4KML4	Costars family protein ABRACL	9030	88.4	46	2	2
Q9JLV5	Cullin-3	88948	128.9	10	5	5
A2A432	Cullin-4B	110699	77.37	6	3	2
Q9CYA0	Cysteine-rich with EGF-like domain protein 2	38220	58.57	8	2	2
Q9ER72	Cysteine--tRNA ligase cytoplasmic	94860	184.38	17	11	11
P56395	Cytochrome b5	15241	82.44	25	2	2
Q9CQX2	Cytochrome b5 type B	16318	145.59	42	3	3
Q9CZ13	Cytochrome b-c1 complex subunit 1 mitochondrial	52852	223.7	33	10	10
Q9DB77	Cytochrome b-c1 complex subunit 2 mitochondrial	48235	206.64	32	9	9
P99028	Cytochrome b-c1 complex subunit 6 mitochondrial	10435	97.21	52	3	3

Q9D855	Cytochrome b-c1 complex subunit 7	13527	88.18	22	2	2
Q8R1I1	Cytochrome b-c1 complex subunit 9	7446	58.69	38	2	2
P62897	Cytochrome c somatic	11605	89.49	39	4	4
P19783	Cytochrome c oxidase subunit 4 isoform 1 mitochondrial	19530	58.31	18	2	2
P12787	Cytochrome c oxidase subunit 5A mitochondrial	16101	78.13	38	4	4
P19536	Cytochrome c oxidase subunit 5B mitochondrial	13813	83.08	23	2	2
P56391	Cytochrome c oxidase subunit 6B1	10071	62.1	29	2	2
Q9D0M3	Cytochrome c1 heme protein mitochondrial	35328	95.01	18	4	3
Q8BKE6	Cytochrome P450 20A1	52149	119.78	17	5	5
O88485	Cytoplasmic dynein 1 intermediate chain 1	70725	127.37	12	4	3
Q8BMK4	Cytoskeleton-associated protein 4	63692	278.87	48	20	20
A2AGT5	Cytoskeleton-associated protein 5	225633	123.69	3	4	4
Q80WW9	DDRGK domain-containing protein 1	35977	93.34	21	3	3
Q99L04	Dehydrogenase/reductase SDR family member 1	34005	96.17	21	4	4
Q99J47	Dehydrogenase/reductase SDR family member 7B	34987	68.44	12	3	3
Q3U9G9	Delta(14)-sterol reductase	71440	120.89	7	3	3
Q9Z110	Delta-1-pyrroline-5-carboxylate synthase	87266	50.55	4	2	2
P82347	Delta-sarcoglycan	32133	81.42	14	3	3
Q3TXU5	Deoxyhypusine synthase	40642	112.4	9	2	2
Q60710	Deoxynucleoside triphosphate triphosphohydrolase SAMHD1	75893	52.6	6	2	2
Q99J56	Derlin-1	28835	67.87	9	2	2
E9Q557	Desmoplakin	332913	78.82	2	4	3
O08749	Dihydrolipoyl dehydrogenase mitochondrial	54272	184.65	22	7	7
Q8BMF4	Dihydrolipoyllysine-residue acetyltransferase component of pyruvate dehydrogenase complex mitochondrial	67942	106.36	5	2	2
Q99KK7	Dipeptidyl peptidase 3	82898	205.75	21	9	9
Q8BPM0	Disheveled-associated activator of morphogenesis 1	123370	68.18	3	2	2
Q99KV1	DnaJ homolog subfamily B member 11	40555	55.5	8	2	2
Q9DC23	DnaJ homolog subfamily C member 10	90583	118.64	13	6	6
O70152	Dolichol-phosphate mannosyltransferase subunit 1	29175	53.96	13	2	2
O54734	Dolichyl-diphosphooligosaccharide--protein glycosyltransferase 48 kDa subunit	49028	200.96	42	12	12
P46978	Dolichyl-diphosphooligosaccharide--protein glycosyltransferase subunit STT3A	80598	173.62	13	8	7

Q3TDQ1	Dolichyl-diphosphooligosaccharide--protein glycosyltransferase subunit STT3B	93246	44.83	4	3	2
Q9QXS6	Drebrin	77287	150.62	11	4	4
P62627	Dynein light chain roadblock-type 1	10990	101.67	29	2	2
Q91ZU6	Dystonin	834234	84.89	1	3	3
Q8BL66	Early endosome antigen 1	160914	110.13	2	2	2
Q6DYE8	Ectonucleotide pyrophosphatase/phosphodiesterase family member 3	98662	177.66	25	14	14
Q9D4J1	EF-hand domain-containing protein D1	27000	63.03	17	2	2
Q9D8Y0	EF-hand domain-containing protein D2	26791	43.59	11	2	2
Q99MS7	EH domain-binding protein 1-like protein 1	184833	66.94	2	2	2
P70372	ELAV-like protein 1	36169	65.86	11	3	3
Q99LC5	Electron transfer flavoprotein subunit alpha mitochondrial	35009	146.13	30	5	5
Q9DCW4	Electron transfer flavoprotein subunit beta	27623	105.08	24	4	4
P62869	Elongin-B	13170	106.75	43	5	5
P83940	Elongin-C	12473	106.91	41	3	3
O08579	Emerin	29436	47.05	10	2	2
Q7TQ95	Endoplasmic reticulum junction formation protein lunapark	47500	36.8	5	2	2
Q3UVK0	Endoplasmic reticulum metallopeptidase 1	100148	163.51	15	7	7
P57759	Endoplasmic reticulum resident protein 29	28823	106.32	22	5	5
Q9D1Q6	Endoplasmic reticulum resident protein 44	46853	166.1	18	5	4
Q9DC16	Endoplasmic reticulum-Golgi intermediate compartment protein 1	32562	89.84	16	3	3
P84089	Enhancer of rudimentary homolog	12259	43.48	27	2	2
P42567	Epidermal growth factor receptor substrate 15	98471	79.93	6	3	3
Q99JH8	ER lumen protein-retaining receptor 1	24560	88.85	25	4	3
Q8C7X2	ER membrane protein complex subunit 1	111605	167.31	17	10	10
Q9CRD2	ER membrane protein complex subunit 2	34935	102.65	16	4	4
Q99KI3	ER membrane protein complex subunit 3	29980	109.4	15	3	3
Q9EP72	ER membrane protein complex subunit 7	26310	144.79	24	4	4
O70378	ER membrane protein complex subunit 8	23348	58.55	17	3	3
Q8R180	ERO1-like protein alpha	54085	112.66	9	2	2
P48024	Eukaryotic translation initiation factor 1	12747	129.83	59	4	4
Q8BMJ3	Eukaryotic translation initiation factor 1A X-chromosomal	16460	70.85	18	2	2
Q3UGC7	Eukaryotic translation initiation factor 3 subunit J-A	29344	105.95	26	4	4

Q66JS6	Eukaryotic translation initiation factor 3 subunit J-B	29486	105.95	26	4	4
Q9DBZ5	Eukaryotic translation initiation factor 3 subunit K	25087	113.84	27	4	4
O35250	Exocyst complex component 7	79960	98.73	8	3	3
Q6PGF7	Exocyst complex component 8	81035	78.36	4	2	2
P54731	FAS-associated factor 1	73863	46.47	5	2	2
Q922J9	Fatty acyl-CoA reductase 1	59435	85.42	9	4	4
Q9QZN4	F-box only protein 6	34492	63.43	13	3	3
Q6P6L0	Filamin A-interacting protein 1-like	129772	50.5	2	2	2
A2AKG8	Focadhesin	198948	64.66	2	2	2
P35505	Fumarylacetoacetase	46176	84.31	10	3	3
Q9JL15	Galectin-8	36162	65.48	8	2	2
Q9QYB5	Gamma-adducin	78777	87.73	6	3	3
O08795	Glucosidase 2 subunit beta	58793	124.18	12	4	4
P26443	Glutamate dehydrogenase 1 mitochondrial	61337	238.92	29	12	12
D3Z7P3	Glutaminase kidney isoform mitochondrial	73964	100.31	9	3	3
Q8BFQ8	Glutamine amidotransferase-like class 1 domain-containing protein 1	23277	69.23	14	2	2
Q80Y14	Glutaredoxin-related protein 5 mitochondrial	16292	75.58	41	3	3
P51855	Glutathione synthetase	52247	82.35	7	3	3
Q64521	Glycerol-3-phosphate dehydrogenase mitochondrial	80954	170.36	19	9	9
Q8CHP8	Glycerol-3-phosphate phosphatase	34541	98.35	9	2	2
Q9D964	Glycine amidinotransferase mitochondrial	48297	168.89	21	6	6
Q8CI94	Glycogen phosphorylase brain form	96730	89.86	7	4	4
Q9R062	Glycogenin-1	37402	50.27	8	3	2
Q9CPV4	Glyoxalase domain-containing protein 4	33317	132.63	23	5	5
Q9R087	Glypican-6	63057	111.41	11	4	4
Q9CRA5	Golgi phosphoprotein 3	33752	119.47	23	4	4
Q6PD26	GPI transamidase component PIG-S	61711	125.68	9	3	3
Q99LP6	GrpE protein homolog 1 mitochondrial	24307	56.49	10	2	2
Q9CQC9	GTP-binding protein SAR1b	22382	85.91	21	3	2
Q9DAS9	Guanine nucleotide-binding protein G(I)/G(S)/G(O) subunit gamma-12	7997	91.2	42	2	2
Q6R0H7	Guanine nucleotide-binding protein G(s) subunit alpha isoforms XLas	121505	249.1	15	13	11
Q9CRB2	H/ACA ribonucleoprotein complex subunit 2	17247	110.48	22	2	2

Q9CQN1	Heat shock protein 75 kDa mitochondrial	80209	179.31	24	9	8
O70252	Heme oxygenase 2	35739	92.45	19	3	3
Q9R257	Heme-binding protein 1	21067	75.29	22	3	3
P17710	Hexokinase-1	108303	181.3	15	12	12
O08528	Hexokinase-2	102535	45.48	3	2	2
P63158	High mobility group protein B1	24894	111.12	34	5	4
P30681	High mobility group protein B2	24162	51.25	20	3	2
Q61035	Histidine--tRNA ligase cytoplasmic	57432	129.4	13	5	5
P43277	Histone H1.3	22100	122.81	11	3	2
P27661	Histone H2AX	15143	171.02	48	6	4
Q61425	Hydroxyacyl-coenzyme A dehydrogenase mitochondrial	34464	77.55	13	2	2
Q9JKR6	Hypoxia up-regulated protein 1	111181	274.18	37	21	21
Q9CR20	Immediate early response 3-interacting protein 1	9017	117.56	55	2	2
O35344	Importin subunit alpha-4	57773	187.76	41	10	9
Q8VI75	Importin-4	119275	92.39	4	3	3
Q9D892	Inosine triphosphate pyrophosphatase	21897	144.18	31	3	3
P50096	Inosine-5'-monophosphate dehydrogenase 1	55279	60.37	10	3	3
O55023	Inositol monophosphatase 1	30436	60.99	11	3	3
P49442	Inositol polyphosphate 1-phosphatase	43346	72.62	6	2	2
Q9JHU9	Inositol-3-phosphate synthase 1	60932	70.73	7	2	2
P15208	Insulin receptor	155610	51.52	5	3	3
Q9JHR7	Insulin-degrading enzyme	117772	57.49	2	2	2
P47877	Insulin-like growth factor-binding protein 2	32847	100.93	19	4	4
Q62470	Integrin alpha-3	116745	289.65	36	26	26
O70309	Integrin beta-5	87909	152.11	15	7	7
Q9Z1X4	Interleukin enhancer-binding factor 3	96021	46.87	3	2	2
Q9D6R2	Isocitrate dehydrogenase [NAD] subunit alpha mitochondrial	39639	74.74	13	3	3
P54071	Isocitrate dehydrogenase [NADP] mitochondrial	50906	159.87	28	11	10
P58044	Isopentenyl-diphosphate Delta-isomerase 1	26289	133.65	25	4	4
Q921I2	Kelch domain-containing protein 4	64861	103.24	5	2	2
Q3UV17	Keratin type II cytoskeletal 2 oral	62845	98.05	10	4	2
Q61595	Kinectin	152592	157.75	9	8	8

E9Q5G3	Kinesin-like protein KIF23	108776	116.76	5	4	4
Q61029	Lamina-associated polypeptide 2 isoforms beta/delta/epsilon/gamma	50373	130.41	19	5	5
P14733	Lamin-B1	66786	184.26	24	10	10
Q8K0C4	Lanosterol 14-alpha demethylase	56776	121.13	30	9	9
Q8BLN5	Lanosterol synthase	83141	69.5	5	3	3
Q6PB66	Leucine-rich PPR motif-containing protein mitochondrial	156614	85.97	4	6	5
Q3UZ39	Leucine-rich repeat flightless-interacting protein 1	79249	120.04	11	4	4
Q9D1G5	Leucine-rich repeat-containing protein 57	26760	97	27	4	4
Q9D154	Leukocyte elastase inhibitor A	42575	123.99	27	7	6
Q61735	Leukocyte surface antigen CD47	33098	108.38	12	3	3
Q61792	LIM and SH3 domain protein 1	29994	76.1	10	2	2
Q9ERG0	LIM domain and actin-binding protein 1	84060	117.91	13	6	6
Q8C3X8	Lipase maturation factor 2	79997	64.98	6	2	2
Q8C8U0	Liprin-beta-1	108540	46.05	3	2	2
Q8CGK3	Lon protease homolog mitochondrial	105843	108.82	9	4	4
P51174	Long-chain specific acyl-CoA dehydrogenase mitochondrial	47908	86.27	6	2	2
Q9ERE7	LRP chaperone MESD	25207	96.75	13	2	2
Q3UN02	Lysocardiolipin acyltransferase 1	44400	80.52	11	2	2
Q8CHK3	Lysophospholipid acyltransferase 7	53436	84.44	10	2	2
P17439	Lysosomal acid glucosylceramidase	57622	86.09	12	3	3
P16675	Lysosomal protective protein	53844	89.32	6	2	2
P17047	Lysosome-associated membrane glycoprotein 2	45681	98.79	9	4	4
Q9DAR7	m7GpppX diphosphatase	38988	139.77	26	6	6
Q60754	Macrophage receptor MARCO	52730	39.12	8	2	2
Q9CQY5	Magnesium transporter protein 1	37970	59.91	7	2	2
Q6ZQI3	Malectin	32342	166.08	25	7	7
Q99M71	Mammalian ependymin-related protein 1	25485	102.33	16	3	3
Q80UM7	Mannosyl-oligosaccharide glucosidase	91831	54.37	4	2	2
P53690	Matrix metalloproteinase-14	65919	129.51	17	8	8
Q9CZH7	Matrix-remodeling-associated protein 7	19458	167.51	36	4	4
P45952	Medium-chain specific acyl-CoA dehydrogenase mitochondrial	46481	115.32	11	2	2
O55022	Membrane-associated progesterone receptor component 1	21694	202.74	43	6	5

Q80UU9	Membrane-associated progesterone receptor component 2	23334	184.52	35	5	4
Q9CXI5	Mesencephalic astrocyte-derived neurotrophic factor	20374	131.46	27	4	4
Q32NY4	Metal transporter CNNM3	76279	80.34	7	2	2
Q99J09	Methylosome protein 50	36943	135.97	30	6	6
Q9QXZ0	Microtubule-actin cross-linking factor 1	831894	157.99	2	11	10
Q8C052	Microtubule-associated protein 1S	102939	67.99	7	4	4
Q9D8V0	Minor histocompatibility antigen H13	41748	128.52	19	4	4
Q9CR62	Mitochondrial 2-oxoglutarate/malate carrier protein	34155	131.94	23	5	5
Q9CQ92	Mitochondrial fission 1 protein	17009	58.75	24	3	3
Q925Q3	Mitochondrial sodium/calcium exchanger protein	64365	53.74	6	2	2
Q9DC61	Mitochondrial-processing peptidase subunit alpha	58279	93.45	5	2	2
Q921Y0	MOB kinase activator 1A	25080	79.22	11	2	2
Q8BPB0	MOB kinase activator 1B	25091	79.22	11	2	2
Q99LR1	Monoacylglycerol lipase ABHD12	45270	85.08	13	4	4
Q9R0E1	Multifunctional procollagen lysine hydroxylase and glycosyltransferase LH3	84922	137.74	12	6	6
Q8K5B2	Multiple coagulation factor deficiency protein 2 homolog	16168	81.15	28	2	2
P97434	Myosin phosphatase Rho-interacting protein	116408	56.3	4	3	3
O08638	Myosin-11	227026	217.7	6	12	2
Q6URW6	Myosin-14	228584	172.51	4	7	2
P62774	Myotrophin	12861	127.07	32	2	2
Q9CWS0	N(G) N(G)-dimethylarginine dimethylaminohydrolase 1	31381	89.9	15	4	3
P70441	Na(+)/H(+) exchange regulatory cofactor NHE-RF1	38600	102.35	14	3	3
Q8BFR4	N-acetylglucosamine-6-sulfatase	61175	107.03	13	5	4
Q8K4Z3	NAD(P)H-hydrate epimerase	30973	99.55	16	3	3
Q99KE1	NAD-dependent malic enzyme mitochondrial	65799	141.71	16	4	4
Q99LC3	NADH dehydrogenase [ubiquinone] 1 alpha subcomplex subunit 10 mitochondrial	40603	84.23	9	2	2
Q7TMF3	NADH dehydrogenase [ubiquinone] 1 alpha subcomplex subunit 12	17086	79.57	20	2	2
Q9DC69	NADH dehydrogenase [ubiquinone] 1 alpha subcomplex subunit 9 mitochondrial	42525	88.56	7	2	2
Q91YT0	NADH dehydrogenase [ubiquinone] flavoprotein 1 mitochondrial	50834	99.21	15	3	3
Q9DCT2	NADH dehydrogenase [ubiquinone] iron-sulfur protein 3 mitochondrial	30149	118.33	21	4	4
Q9DC70	NADH dehydrogenase [ubiquinone] iron-sulfur protein 7 mitochondrial	24683	102.03	20	2	2
Q91VD9	NADH-ubiquinone oxidoreductase 75 kDa subunit mitochondrial	79777	127.29	15	6	6

P03921	NADH-ubiquinone oxidoreductase chain 5	68475	91.34	5	2	2
P37040	NADPH--cytochrome P450 reductase	77044	151.43	21	8	8
Q8BWZ3	N-alpha-acetyltransferase 25 NatB auxiliary subunit	111708	71.39	5	2	2
Q8BHG1	Nardilysin	132891	153.1	14	10	10
Q8VBW6	NEDD8-activating enzyme E1 regulatory subunit	60274	76.85	11	3	3
Q91YP2	Neurolysin mitochondrial	80429	63.39	8	4	3
Q8BNY6	Neuronal calcium sensor 1	21879	142.14	43	6	6
Q8BHN3	Neutral alpha-glucosidase AB	106911	231.62	31	23	23
Q8BLF1	Neutral cholesterol ester hydrolase 1	45740	134.83	20	4	4
Q99KQ4	Nicotinamide phosphoribosyltransferase	55447	46.25	7	2	2
Q6GQT9	Nodal modulator 1	133420	192.37	23	16	16
Q99P88	Nuclear pore complex protein Nup155	155117	74.65	3	2	2
P60670	Nuclear protein localization protein 4 homolog	68017	79.18	12	3	3
P61971	Nuclear transport factor 2	14478	54.25	34	2	2
Q02819	Nucleobindin-1	53409	120.11	12	4	4
P81117	Nucleobindin-2	50305	41.22	5	2	2
E9Q5C9	Nucleolar and coiled-body phosphoprotein 1	73698	62.4	4	2	2
Q9D1X0	Nucleolar protein 3	24568	134.75	29	3	3
P97346	Nucleoredoxin	48344	68.55	6	2	2
P15532	Nucleoside diphosphate kinase A	17208	210.39	62	9	4
Q6PIP5	NudC domain-containing protein 1	66705	79.17	4	2	2
Q9JHW2	Omega-amidase NIT2	30502	73.87	12	2	2
P29758	Ornithine aminotransferase mitochondrial	48355	224.41	45	12	12
Q62422	Osteoclast-stimulating factor 1	23783	91.73	14	2	2
O54901	OX-2 membrane glycoprotein	31256	57.02	8	2	2
Q8CHP5	Partner of Y14 and mago	22690	60.13	13	2	2
Q99JF8	PC4 and SFRS1-interacting protein	59697	78.87	4	2	2
O70400	PDZ and LIM domain protein 1	35774	136.36	32	6	6
P24369	Peptidyl-prolyl cis-trans isomerase B	23713	170.03	41	10	10
Q61576	Peptidyl-prolyl cis-trans isomerase FKBP10	64698	107.82	8	4	4
Q9D1M7	Peptidyl-prolyl cis-trans isomerase FKBP11	22137	102.04	31	4	4
P45878	Peptidyl-prolyl cis-trans isomerase FKBP2	15344	150.3	39	3	3

Q62446	Peptidyl-prolyl cis-trans isomerase FKBP3	25148	56.15	12	2	2
Q9Z247	Peptidyl-prolyl cis-trans isomerase FKBP9	62996	89.02	10	4	4
Q8R2Y8	Peptidyl-tRNA hydrolase 2 mitochondrial	19527	153.82	35	4	4
O08807	Peroxiredoxin-4	31053	205.25	49	10	8
Q9DC50	Peroxisomal carnitine O-octanoyltransferase	70264	70.07	5	2	2
Q9R0A0	Peroxisomal membrane protein PEX14	41208	93.56	22	4	4
Q501J7	Phosphatase and actin regulator 4	76632	135.29	11	5	5
Q8VEM8	Phosphate carrier protein mitochondrial	39632	186.29	36	11	11
Q99L43	Phosphatidate cytidylyltransferase 2	51314	100.98	8	2	2
Q9EP69	Phosphatidylinositide phosphatase SAC1	66944	43.63	4	2	2
P53810	Phosphatidylinositol transfer protein alpha isoform	31893	126.47	20	3	3
P53811	Phosphatidylinositol transfer protein beta isoform	31487	67.35	17	3	3
Q7TSV4	Phosphoglucomutase-2	68748	51.88	7	2	2
P27612	Phospholipase A-2-activating protein	87221	149.58	14	6	6
Q99JY8	Phospholipid phosphatase 3	35216	42.13	10	2	2
E2JF22	Piezo-type mechanosensitive ion channel component 1	292000	76.94	1	2	2
P70207	Plexin-A2	211533	53.24	2	2	2
Q9Z1W8	Potassium-transporting ATPase alpha chain 2	114726	121.91	6	6	2
Q9JIG8	PRA1 family protein 2	19478	85.84	16	2	2
Q3TVI8	Pre-B-cell leukemia transcription factor-interacting protein 1	81165	138.66	15	6	6
P61759	Prefoldin subunit 3	22436	132.8	21	3	3
Q9WU28	Prefoldin subunit 5	17356	139.28	36	4	4
Q03958	Prefoldin subunit 6	14455	105.85	31	3	3
Q9CQF9	Prenylcysteine oxidase	56495	138.18	11	3	3
Q8C7K6	Prenylcysteine oxidase-like	54875	83.8	8	3	3
Q9R0E2	Procollagen-lysine 2-oxoglutarate 5-dioxygenase 1	83595	125.99	12	6	6
Q9R0B9	Procollagen-lysine 2-oxoglutarate 5-dioxygenase 2	84488	190.59	22	11	11
Q8VE70	Programmed cell death protein 10	24716	38.9	11	2	2
P56812	Programmed cell death protein 5	14275	93.65	37	4	4
P28798	Progranulin	63458	91.78	5	2	2
P67778	Prohibitin	29820	214.96	71	13	13
O35129	Prohibitin-2	33296	196.83	49	13	13

Q9WUQ2	Prolactin regulatory element-binding protein	45437	52.12	6	2	2
Q3V1T4	Prolyl 3-hydroxylase 1	83651	86.94	4	2	2
Q60716	Prolyl 4-hydroxylase subunit alpha-2	61002	123.58	16	5	5
O35074	Prostacyclin synthase	57047	137.3	20	7	7
Q9R0Q7	Prostaglandin E synthase 3	18721	158.15	31	4	4
P22437	Prostaglandin G/H synthase 1	69042	184.21	31	11	11
Q9JKV1	Proteasomal ubiquitin receptor ADRM1	42060	74.43	5	2	2
P97371	Proteasome activator complex subunit 1	28673	123.44	23	3	3
P97372	Proteasome activator complex subunit 2	27057	119.5	16	2	2
P49722	Proteasome subunit alpha type-2	25927	230.6	46	9	9
O55234	Proteasome subunit beta type-5	28532	196.35	35	10	10
Q91VR8	Protein BRICK1	8761	38.4	25	2	2
Q9QXT0	Protein canopy homolog 2	20767	104.84	23	3	3
Q8BQ47	Protein canopy homolog 4	28094	59.66	17	2	2
Q4VAA2	Protein CDV3	29729	116.98	32	5	5
Q9CQ89	Protein CutA	18865	78.49	18	2	2
O08808	Protein diaphanous homolog 1	139343	83.28	6	3	3
P08003	Protein disulfide-isomerase A4	71982	257.7	39	21	21
Q921X9	Protein disulfide-isomerase A5	59267	63.27	5	2	2
Q9D0F3	Protein ERGIC-53	57789	173.65	24	6	6
Q8VE88	Protein FAM114A2	54045	78.42	8	3	3
Q8BYI8	Protein FAM234B	67031	94.7	12	3	3
Q61239	Protein farnesyltransferase/geranylgeranyltransferase type-1 subunit alpha	44013	104.82	13	3	3
Q9CR64	Protein kish-A	8074	58.62	25	2	2
Q8JZS0	Protein lin-7 homolog A	25993	81.96	11	2	2
O88952	Protein lin-7 homolog C	21834	81.96	13	2	2
Q80WJ7	Protein LYRIC	63846	152.07	12	7	7
Q8K3C3	Protein LZIC	21537	111.38	17	2	2
Q9JHP7	Protein O-glucosyltransferase 2	57985	94.29	9	3	3
Q9D0B6	Protein PBDC1	22223	143.1	43	6	6
Q9DBR7	Protein phosphatase 1 regulatory subunit 12A	114996	60.96	3	2	2
Q62084	Protein phosphatase 1 regulatory subunit 14B	15957	147.13	69	7	7

Q61074	Protein phosphatase 1G	58728	59.24	11	2	2
Q9DCL8	Protein phosphatase inhibitor 2	23119	102.01	9	2	2
Q3UPH1	Protein PRRC1	46298	110.52	10	2	2
Q7TSC1	Protein PRRC2A	229200	65.54	3	2	2
Q3TLH4	Protein PRRC2C	310891	75.8	3	4	4
Q9Z2G6	Protein sel-1 homolog 1	88340	62.29	7	3	3
Q9EQU5	Protein SET	33378	167.01	35	6	6
Q9CX34	Protein SGT1 homolog	38159	191.3	29	6	6
Q9D662	Protein transport protein Sec23B	86437	200.92	19	10	7
P61620	Protein transport protein Sec61 subunit alpha isoform 1	52265	224.28	36	11	7
Q6DID7	Protein wntless homolog	62188	53.88	7	3	3
Q9EPK2	Protein XRP2	39377	90.67	9	2	2
Q99LX0	Protein/nucleic acid deglycase DJ-1	20021	142.43	26	4	4
P26350	Prothymosin alpha	12254	157.22	41	6	6
Q8K183	Pyridoxal kinase	35015	95.13	12	2	2
Q9DCC4	Pyrroline-5-carboxylate reductase 3	28721	85.07	11	2	2
P52480	Pyruvate kinase PKM	57845	398.65	66	39	38
P47199	Quinone oxidoreductase	35269	75.26	11	2	2
P50396	Rab GDP dissociation inhibitor alpha	50522	279.68	46	15	9
Q9WVM1	Rac GTPase-activating protein 1	70159	72.14	5	2	2
Q9JHS3	Ragulator complex protein LAMTOR2	13480	68.29	33	3	3
O88653	Ragulator complex protein LAMTOR3	13553	89.75	44	3	3
Q9D1L9	Ragulator complex protein LAMTOR5	9642	128.07	44	2	2
Q9CT10	Ran-binding protein 3	52573	99.09	6	2	2
P97379	Ras GTPase-activating protein-binding protein 2	54088	80.85	8	2	2
Q99P58	Ras-related protein Rab-27B	24560	104.92	18	3	2
Q921E2	Ras-related protein Rab-31	21331	99.53	14	2	2
P55258	Ras-related protein Rab-8A	23668	116.26	23	4	2
P18052	Receptor-type tyrosine-protein phosphatase alpha	93698	60.37	4	2	2
Q05186	Reticulocalbin-1	38113	146.54	18	5	5
Q8BP92	Reticulocalbin-2	37271	148.05	27	5	5
Q9ES97	Reticulon-3	103879	161.98	6	5	5

Q9QYF1	Retinol dehydrogenase 11	35148	60.3	8	2	2
Q61599	Rho GDP-dissociation inhibitor 2	22851	113.57	36	3	3
Q5FWK3	Rho GTPase-activating protein 1	50411	181.11	32	10	10
Q91VI7	Ribonuclease inhibitor	49817	278.24	65	18	18
P11157	Ribonucleoside-diphosphate reductase subunit M2	45096	65.78	6	2	2
Q9JI75	Ribosyldihydronicotinamide dehydrogenase [quinone]	26248	99.35	15	2	2
Q91VM5	RNA binding motif protein X-linked-like-1	42162	75.81	10	3	3
Q9D706	RNA polymerase II-associated protein 3	74096	49.98	5	2	2
Q9WV02	RNA-binding motif protein X chromosome	42301	75.81	10	3	3
O89086	RNA-binding protein 3	16605	105.08	30	2	2
Q61545	RNA-binding protein EWS	68462	65.34	4	2	2
Q9CQK7	RWD domain-containing protein 1	27785	102.36	17	2	2
O55143	Sarcoplasmic/endoplasmic reticulum calcium ATPase 2	114858	234.95	20	15	9
Q80YR5	Scaffold attachment factor B2	111839	91.88	6	3	3
Q8CFE4	SCY1-like protein 2	103317	108.17	8	4	4
Q8BRF7	Sec1 family domain-containing protein 1	72323	68.13	6	2	2
Q6NZC7	SEC23-interacting protein	110780	62.66	8	4	4
Q9ERN0	Secretory carrier-associated membrane protein 2	36465	84.11	10	2	2
Q9R1T4	Septin-6	49620	118.51	22	6	2
Q8CHH9	Septin-8	49812	166.44	26	7	4
P97363	Serine palmitoyltransferase 2	62982	142.82	15	5	5
O35326	Serine/arginine-rich splicing factor 5	30891	63.09	10	2	2
Q99KH8	Serine/threonine-protein kinase 24	47954	130.47	18	4	4
O88643	Serine/threonine-protein kinase PAK 1	60737	81.17	10	4	3
Q5F2E8	Serine/threonine-protein kinase TAO1	116050	73.85	3	3	3
Q60996	Serine/threonine-protein phosphatase 2A 56 kDa regulatory subunit gamma isoform	60825	77.84	6	2	2
Q7TNP2	Serine/threonine-protein phosphatase 2A 65 kDa regulatory subunit A beta isoform	65934	195.45	18	8	2
P58389	Serine/threonine-protein phosphatase 2A activator	36710	100.48	12	2	2
Q62087	Serum paraoxonase/lactonase 3	39351	98.98	15	3	3
Q9R0P3	S-formylglutathione hydrolase	31320	263.32	70	10	10
Q8R550	SH3 domain-containing kinase-binding protein 1	78170	104.93	5	3	3
Q99JR1	Sideroflexin-1	35649	57.75	12	2	2

Q91V61	Sideroflexin-3	35406	128.42	19	3	3
O55242	Sigma non-opioid intracellular receptor 1	25250	102.48	11	2	2
Q9R0P6	Signal peptidase complex catalytic subunit SEC11A	20626	44.95	11	2	2
Q9CYN2	Signal peptidase complex subunit 2	24978	66.38	16	2	2
Q6ZWQ7	Signal peptidase complex subunit 3	20313	93.1	28	4	4
Q9JJF9	Signal peptide peptidase-like 2A	58129	104.03	5	2	2
P16254	Signal recognition particle 14 kDa protein	12510	60.99	24	2	2
P47758	Signal recognition particle receptor subunit beta	29579	160.66	25	5	5
Q8BJU0	Small glutamine-rich tetratricopeptide repeat-containing protein alpha	34322	57.6	11	3	3
P14094	Sodium/potassium-transporting ATPase subunit beta-1	35195	117.93	16	4	4
Q8K2P7	Sodium-coupled neutral amino acid transporter 1	53795	92.31	7	3	3
Q9EPR4	Solute carrier family 23 member 2	70049	180.09	19	7	7
Q64442	Sorbitol dehydrogenase	38249	49.39	9	2	2
P07214	SPARC	34450	130.77	17	5	5
Q8R1X6	Spartin	72655	58.98	4	2	2
O09005	Sphingolipid delta(4)-desaturase DES1	38241	104.33	16	3	3
O08530	Sphingosine 1-phosphate receptor 1	42639	46.79	6	2	2
Q8R0X7	Sphingosine-1-phosphate lyase 1	63677	126.23	11	5	5
Q9Z1N5	Spliceosome RNA helicase Ddx39b	49035	131.9	21	10	3
Q9D883	Splicing factor U2AF 35 kDa subunit	27815	59.05	13	2	2
P26369	Splicing factor U2AF 65 kDa subunit	53517	82.29	7	3	3
P52019	Squalene monooxygenase	63770	93.51	5	2	2
Q8C7E7	Starch-binding domain-containing protein 1	36127	56.61	12	2	2
Q99JB2	Stomatin-like protein 2 mitochondrial	38385	68.68	9	2	2
O55106	Striatin	85966	74.65	6	3	3
Q9ERG2	Striatin-3	87150	36.54	4	2	2
Q8K2B3	Succinate dehydrogenase [ubiquinone] flavoprotein subunit mitochondrial	72585	190.82	23	9	9
Q9D0K2	Succinyl-CoA:3-ketoacid coenzyme A transferase 1 mitochondrial	55989	189.18	36	8	8
Q62273	Sulfate transporter	81604	45.19	3	2	2
P09671	Superoxide dismutase [Mn] mitochondrial	24603	127.48	19	3	3
Q64310	Surfeit locus protein 4	30381	124.05	16	3	3
Q9CQW1	Synaptobrevin homolog YKT6	22314	116.96	24	4	4

Q3TIR3	Synembryn-A	59847	58.88	5	2	2
Q00262	Syntaxin-2	33178	59.84	16	2	2
O70439	Syntaxin-7	29821	95.63	20	3	3
Q64324	Syntaxin-binding protein 2	66358	86.54	7	2	2
Q9R233	Tapasin	49736	36.24	5	2	2
P97493	Thioredoxin mitochondrial	18255	120.13	42	4	4
Q9CQU0	Thioredoxin domain-containing protein 12	19049	93.58	14	2	2
Q9CQM5	Thioredoxin domain-containing protein 17	14015	89.63	27	3	3
P20108	Thioredoxin-dependent peroxide reductase mitochondrial	28127	152.14	33	6	6
Q8VBT0	Thioredoxin-related transmembrane protein 1	31396	98.26	18	4	4
Q9D710	Thioredoxin-related transmembrane protein 2	33943	80.28	9	2	2
O08583	THO complex subunit 4	26940	96.8	15	2	2
P20352	Tissue factor	32935	89.45	9	2	2
Q80ZI1	TMF-regulated nuclear protein 1	23086	36.26	16	2	2
Q9ER41	Torsin-1B	37818	86.4	9	2	2
P37804	Transgelin	22576	83.51	21	4	4
Q7TN37	Transient receptor potential cation channel subfamily M member 4	135760	40.32	2	2	2
Q8VHE0	Translocation protein SEC63 homolog	87870	152.11	16	7	7
P50637	Translocator protein	18841	151.64	37	4	4
Q9CY50	Translocon-associated protein subunit alpha	32065	122.01	18	4	4
Q62186	Translocon-associated protein subunit delta	18937	116.87	25	3	3
Q9DCF9	Translocon-associated protein subunit gamma	21064	118.98	22	2	2
Q3V009	Transmembrane emp24 domain-containing protein 1	25263	87.42	15	2	2
Q9R0Q3	Transmembrane emp24 domain-containing protein 2	22705	101.11	25	3	3
Q9CXE7	Transmembrane emp24 domain-containing protein 5	26172	89.15	13	2	2
Q99KF1	Transmembrane emp24 domain-containing protein 9	27127	77.41	20	4	3
Q9D771	Transmembrane protein 206	40178	66.41	14	2	2
Q9CR67	Transmembrane protein 33	28031	73.69	9	2	2
Q9DBS1	Transmembrane protein 43	44783	181.43	32	8	8
Q8BXN9	Transmembrane protein 87A	63380	71.18	6	2	2
Q8BI84	Transport and Golgi organization protein 1 homolog	213673	63.29	2	2	2
O89023	Tripeptidyl-peptidase 1	61342	93.81	10	3	3

Q9JHJ0	Tropomodulin-3	39503	47.8	9	2	2
P58771	Tropomyosin alpha-1 chain	32681	265.66	38	16	5
P58774	Tropomyosin beta chain	32837	210.5	43	12	7
Q9CWF2	Tubulin beta-2B chain	49953	312.75	72	23	4
Q8VCK3	Tubulin gamma-2 chain	51122	77.1	12	3	3
Q9QZM4	Tumor necrosis factor receptor superfamily member 10B	42165	71.13	8	2	2
Q62393	Tumor protein D52	24313	71.66	14	2	2
Q9CYZ2	Tumor protein D54	24043	113.54	29	5	5
P35235	Tyrosine-protein phosphatase non-receptor type 11	68460	111.89	14	5	5
Q91WQ3	Tyrosine--tRNA ligase cytoplasmic	59105	147.92	13	5	5
O35900	U6 snRNA-associated Sm-like protein LSm2	10835	64.98	28	2	2
Q6ZWM4	U6 snRNA-associated Sm-like protein LSm8	10403	113.64	52	3	3
Q8R317	Ubiquilin-1	61976	144.86	11	4	2
Q9QZM0	Ubiquilin-2	67351	162.31	9	4	2
Q9WUP7	Ubiquitin carboxyl-terminal hydrolase isozyme L5	37617	48.83	7	2	2
P70362	Ubiquitin recognition factor in ER-associated degradation protein 1	34481	51.66	10	2	2
Q80X50	Ubiquitin-associated protein 2-like	116799	69.25	3	2	2
Q9D2M8	Ubiquitin-conjugating enzyme E2 variant 2	16367	74.01	20	3	3
Q8VCH8	UBX domain-containing protein 4	56472	113.14	8	2	2
Q6P5E4	UDP-glucose:glycoprotein glucosyltransferase 1	176433	215.29	21	23	23
A2A7S8	Uncharacterized protein KIAA1522	104842	81.58	3	2	2
P54726	UV excision repair protein RAD23 homolog A	39707	113.81	19	3	2
P54728	UV excision repair protein RAD23 homolog B	43513	182.7	48	12	11
Q9QZ88	Vacuolar protein sorting-associated protein 29	20496	40.99	12	2	2
Q99KU0	Vacuole membrane protein 1	45960	50.6	11	2	2
P50544	Very long-chain specific acyl-CoA dehydrogenase mitochondrial	70876	102.54	12	5	5
O70503	Very-long-chain 3-oxoacyl-CoA reductase	34742	176.72	49	12	12
O70404	Vesicle-associated membrane protein 8	11451	86.52	14	2	2
Q9WV55	Vesicle-associated membrane protein-associated protein A	27855	168.59	44	9	8
Q9QY76	Vesicle-associated membrane protein-associated protein B	26946	67.66	14	3	2
P46460	Vesicle-fusing ATPase	82613	58.39	5	3	3
O08547	Vesicle-trafficking protein SEC22b	24741	90.48	15	2	2

Q60931	Voltage-dependent anion-selective channel protein 3	30753	101.73	15	3	2
Q66JT1	Volume-regulated anion channel subunit LRRC8E	90501	40.48	4	2	2
Q99KC8	von Willebrand factor A domain-containing protein 5A	87143	52.06	5	3	3
Q9Z1G3	V-type proton ATPase subunit C 1	43888	91.31	14	2	2
Q9D1K2	V-type proton ATPase subunit F	13370	93.98	30	2	2
Q9Z2C8	Y-box-binding protein 2	38271	174.77	27	9	5
Q3TIV5	Zinc finger CCCH domain-containing protein 15	48327	90.62	7	2	2
Q31125	Zinc transporter SLC39A7	50657	103.47	9	2	2
Q6P5F6	Zinc transporter ZIP10	94394	103.64	4	2	2
Q8C145	Zinc transporter ZIP6	86380	62.89	3	2	2

Table S4. Proteins exclusively present in Large Evs. For each protein Acc. No, description, -10lgP, sequence coverage, the number of identified peptides and the number of unique peptides are reported.

Accession	Description	Avg. Mass	-10lgP	Coverage (%)	Peptides	Unique peptides
Q8K2J0	1-phosphatidylinositol 4 5-bisphosphate phosphodiesterase delta-3	88607	144.07	14	5	5
Q99J99	3-mercaptopyruvate sulfurtransferase	33097	54.74	15	2	2
Q6ZWY3	40S ribosomal protein S27-like	9477	125.76	39	4	2
Q5EG47	5'-AMP-activated protein kinase catalytic subunit alpha-1	63929	117.17	7	3	3
P53026	60S ribosomal protein L10a	24916	199.18	38	10	10
O55142	60S ribosomal protein L35a	12554	51.79	15	3	3
Q5SWU9	Acetyl-CoA carboxylase 1	265254	206.43	12	19	19
Q9Z2N8	Actin-like protein 6A	47448	76.44	16	4	4
P55264	Adenosine kinase	40149	131.13	19	4	4
Q640N1	Adipocyte enhancer-binding protein 1	128365	67.38	3	2	2
Q9CQW2	ADP-ribosylation factor-like protein 8B	21539	140.94	51	8	3
Q8CG76	Aflatoxin B1 aldehyde reductase member 2	40612	108.8	11	3	3
Q9DBR0	A-kinase anchor protein 8	76294	47.46	9	2	2
Q6GQT1	Alpha-2-macroglobulin-P	164352	172.89	3	4	3
P00687	Alpha-amylase 1	57644	37.94	4	2	2
Q91W96	Anaphase-promoting complex subunit 4	91708	74.63	5	3	3
Q9WVM3	Anaphase-promoting complex subunit 7	63021	105.41	7	2	2
P32261	Antithrombin-III	52004	88.15	10	3	3
E9Q414	Apolipoprotein B-100	509437	58.93	1	2	2
Q99KN1	Arrestin domain-containing protein 1	46330	189.14	40	8	8
Q99LE6	ATP-binding cassette sub-family F member 2	71782	42.24	4	2	2
P47857	ATP-dependent 6-phosphofructokinase muscle type	85269	67.29	7	4	3
Q9WUA3	ATP-dependent 6-phosphofructokinase platelet type	85455	187.7	20	11	9
Q8K363	ATP-dependent RNA helicase DDX18	74181	118.57	13	5	5
Q9ESV0	ATP-dependent RNA helicase DDX24	96429	60.1	4	2	2
Q8VDW0	ATP-dependent RNA helicase DDX39A	49067	166.43	27	11	5
Q6PGC1	ATP-dependent RNA helicase DHX29	153975	163.33	9	8	8
Q99PU8	ATP-dependent RNA helicase DHX30	136668	70.39	2	2	2

P56485	Atypical chemokine receptor 3	41636	113.17	7	2	2
Q9Z2H5	Band 4.1-like protein 1	98315	142.57	8	6	5
O54962	Barrier-to-autointegration factor	10103	123.12	47	4	4
Q05793	Basement membrane-specific heparan sulfate proteoglycan core protein	398295	453.37	37	84	84
Q8K019	Bcl-2-associated transcription factor 1	106002	50.43	4	2	2
P20060	Beta-hexosaminidase subunit beta	61116	57.82	6	2	2
P98063	Bone morphogenetic protein 1	111666	81.52	6	4	4
P36895	Bone morphogenetic protein receptor type-1A	60063	69.6	7	2	2
Q8K3W0	BRISC and BRCA1-A complex member 2	43545	59.57	7	2	2
P33146	Cadherin-15	85644	73.6	4	2	2
P15116	Cadherin-2	99796	118.48	5	3	3
Q923T9	Calcium/calmodulin-dependent protein kinase type II subunit gamma	59607	98.25	15	5	4
O08688	Calpain-5	72955	57.05	6	2	2
P48758	Carbonyl reductase [NADPH] 1	30641	62.17	13	3	2
Q6ZQ08	CCR4-NOT transcription complex subunit 1	266805	82.1	2	4	4
Q8VDP4	Cell cycle and apoptosis regulator protein 2	103002	83.8	7	3	3
Q8BGZ4	Cell division cycle protein 23 homolog	68562	72.19	5	2	2
Q99M54	Cell division cycle-associated protein 3	28708	48.15	13	2	2
O54692	Centromere/kinetochore protein zw10 homolog	88063	149.37	11	5	5
O88693	Ceramide glucosyltransferase	44839	206.38	34	10	10
O35454	Chloride transport protein 6	96980	88.17	4	2	2
Q8VHY0	Chondroitin sulfate proteoglycan 4	252305	105.65	4	4	4
Q6PDQ2	Chromodomain-helicase-DNA-binding protein 4	217749	95.95	2	3	3
Q9EPU4	Cleavage and polyadenylation specificity factor subunit 1	160818	106.33	4	3	3
Q5SW19	Clustered mitochondria protein homolog	148068	108.81	9	7	7
O88783	Coagulation factor V	247228	127.59	2	3	3
Q9JIF7	Coatomer subunit beta	107066	235.25	27	15	15
P28481	Collagen alpha-1(II) chain	141974	55.15	6	4	3
Q04857	Collagen alpha-1(VI) chain	108489	48.32	4	2	2
Q60847	Collagen alpha-1(XII) chain	340215	174.32	7	12	12
P39061	Collagen alpha-1(XVIII) chain	182171	107.98	6	5	5
Q2UY11	Collagen alpha-1(XXVIII) chain	118749	47.03	7	3	3

Q3U962	Collagen alpha-2(V) chain	145018	65.46	3	3	3
P01027	Complement C3	186483	159.06	4	7	6
P59108	Copine-2	61036	73.54	5	2	2
Q9DC53	Copine-8	64667	86.98	9	4	4
Q9QZQ8	Core histone macro-H2A.1	39735	166.44	34	8	6
P45481	CREB-binding protein	265491	51.73	2	2	2
Q91YT8	CSC1-like protein 1	91860	72.19	5	3	3
Q5SUQ9	CST complex subunit CTC1	134028	62.69	4	2	2
P97314	Cysteine and glycine-rich protein 2	20926	81.14	15	2	2
Q8BGE6	Cysteine protease ATG4B	44375	84.08	13	3	3
Q9D1A2	Cytosolic non-specific dipeptidase	52767	78.34	4	2	2
Q3V1L4	Cytosolic purine 5'-nucleotidase	64809	52.38	6	2	2
A2AF47	Dedicator of cytokinesis protein 11	237769	50.68	2	2	2
Q9QXB9	Developmentally-regulated GTP-binding protein 2	40718	188.43	29	8	8
Q91ZV3	Discoidin CUB and LCCL domain-containing protein 2	83774	86.58	6	2	2
Q3USZ8	Divergent protein kinase domain 2A	49463	69.6	7	2	2
P37913	DNA ligase 1	102290	69.58	5	3	3
P25206	DNA replication licensing factor MCM3	91546	216.56	24	9	9
P49717	DNA replication licensing factor MCM4	96736	164.89	18	13	13
P49718	DNA replication licensing factor MCM5	82343	130.38	11	6	6
P97311	DNA replication licensing factor MCM6	92867	181.07	17	10	10
Q61881	DNA replication licensing factor MCM7	81211	267.44	34	16	16
Q04750	DNA topoisomerase 1	90876	109.95	8	4	4
Q64511	DNA topoisomerase 2-beta	181908	132.2	8	8	5
P08775	DNA-directed RNA polymerase II subunit RPB1	217174	158.83	5	7	7
Q8CFI7	DNA-directed RNA polymerase II subunit RPB2	133910	139.68	8	6	6
Q9QYJ3	DnaJ homolog subfamily B member 1	38167	69.15	9	3	2
Q9D832	DnaJ homolog subfamily B member 4	37782	66.89	12	3	2
P51807	Dynein light chain Tctex-type 1	12483	50.12	37	2	2
Q3U487	E3 ubiquitin-protein ligase HECTD3	97347	60.76	3	2	2
Q8C863	E3 ubiquitin-protein ligase Itchy	98994	106.02	10	5	4
Q80UY2	E3 ubiquitin-protein ligase KCMF1	41791	99.24	8	2	2

Q8CFI0	E3 ubiquitin-protein ligase NEDD4-like	115419	170.31	10	9	2
Q3U2C5	E3 ubiquitin-protein ligase RNF149	42553	94.18	11	3	3
E9Q555	E3 ubiquitin-protein ligase RNF213	584791	156.76	2	8	8
Q8C0E3	E3 ubiquitin-protein ligase TRIM47	69912	231.55	29	11	11
G5E870	E3 ubiquitin-protein ligase TRIP12	224126	68.41	2	3	3
Q80TP3	E3 ubiquitin-protein ligase UBR5	308351	40.71	2	2	2
Q80TA9	Ectopic P granules protein 5 homolog	290799	67.82	1	2	2
Q9QXY6	EH domain-containing protein 3	60821	220.85	28	15	2
P54320	Elastin	71938	53.74	2	2	2
Q8JZR6	Electroneutral sodium bicarbonate exchanger 1	122421	60.45	3	2	2
Q7TT37	Elongator complex protein 1	149583	184.24	10	8	8
Q91WG4	Elongator complex protein 2	93093	74.18	5	2	2
Q9CZX0	Elongator complex protein 3	62385	114.33	10	4	4
Q99K41	EMILIN-1	107585	164.84	12	7	7
Q8K482	EMILIN-2	117310	103.04	3	2	2
Q8BHL5	Engulfment and cell motility protein 2	83887	57.88	7	3	3
P54754	Ephrin type-B receptor 3	109662	141.64	7	5	2
P54761	Ephrin type-B receptor 4	108848	135.53	7	5	2
Q8R0W0	Epiplakin	724747	128.3	1	5	2
Q9CQM2	ER lumen protein-retaining receptor 2	24454	61.77	16	2	2
Q91VC3	Eukaryotic initiation factor 4A-III	46840	195.75	31	10	7
Q9D1M4	Eukaryotic translation elongation factor 1 epsilon-1	19859	49.88	20	2	2
Q9DBZ5	Eukaryotic translation initiation factor 3 subunit K	25087	95.52	13	2	2
Q62448	Eukaryotic translation initiation factor 4 gamma 2	102105	60.69	5	3	3
Q8BMB3	Eukaryotic translation initiation factor 4E type 2	28263	43.21	12	2	2
O35382	Exocyst complex component 4	110545	72.54	3	2	2
Q8BTW3	Exosome complex component MTR3	28370	62.84	15	2	2
Q9CSH3	Exosome complex exonuclease RRP44	108837	71.09	6	3	3
Q9CZU3	Exosome RNA helicase MTR4	117636	46.12	3	2	2
Q9EPK7	Exportin-7	123810	122.74	7	4	4
Q8K007	Extracellular sulfatase Sulf-1	100923	87.08	6	2	2
Q920B9	FACT complex subunit SPT16	119825	130.73	11	7	7

Q08943	FACT complex subunit SSRP1	80860	85.92	7	4	4
Q6P9Q4	FH1/FH2 domain-containing protein 1	129600	61.88	3	2	2
Q08879	Fibulin-1	78033	68.93	6	2	2
P37889	Fibulin-2	131834	270.79	27	20	20
Q9R0N0	Galactokinase	42295	174.37	33	8	8
Q07797	Galectin-3-binding protein	64491	218.25	28	9	9
Q9CWZ7	Gamma-soluble NSF attachment protein	34732	81.75	10	2	2
Q921G8	Gamma-tubulin complex component 2	103223	128.6	6	4	4
P58854	Gamma-tubulin complex component 3	103469	148.71	9	6	6
Q8BKN5	Gamma-tubulin complex component 5	117977	93.29	4	2	2
Q8BX17	Gem-associated protein 5	166591	57.17	2	2	2
Q8BMQ2	General transcription factor 3C polypeptide 4	91613	66.7	4	2	2
Q9Z1E4	Glycogen [starch] synthase muscle	83927	136.94	10	4	4
Q9WV60	Glycogen synthase kinase-3 beta	46710	192.35	30	7	7
O70311	Glycylpeptide N-tetradecanoyltransferase 2	60484	119.98	12	4	2
Q99L27	GMP reductase 2	38019	95.36	7	2	2
Q8BXA1	Golgi integral membrane protein 4	76785	74.96	7	3	3
O70326	Gremlin-1	20710	97.86	15	2	2
P43029	Growth/differentiation factor 7	47891	52.79	7	2	2
Q6R0H7	Guanine nucleotide-binding protein G(s) subunit alpha isoforms XLas	121505	219.58	16	15	13
Q8CI11	Guanine nucleotide-binding protein-like 3	60787	166.68	18	5	5
Q9CY66	H/ACA ribonucleoprotein complex subunit 1	23474	54.15	11	3	3
Q99M31	Heat shock 70 kDa protein 14	54650	68.67	8	2	2
P48722	Heat shock 70 kDa protein 4L	94382	103.58	5	3	2
P17156	Heat shock-related 70 kDa protein 2	69642	251.61	24	13	3
Q7TN16	Hedgehog-interacting protein	78513	49.94	4	2	2
P01942	Hemoglobin subunit alpha	15085	96.78	17	2	2
Q3TEA8	Heterochromatin protein 1-binding protein 3	60867	115.77	14	4	4
Q60668	Heterogeneous nuclear ribonucleoprotein D0	38354	121.67	16	3	3
Q921F4	Heterogeneous nuclear ribonucleoprotein L-like	64125	77.56	9	3	3
Q09143	High affinity cationic amino acid transporter 1	67092	136.08	10	3	3
O09106	Histone deacetylase 1	55075	99.75	12	3	2

O88895	Histone deacetylase 3	48364	39.26	7	2	2
P43274	Histone H1.4	21977	199.12	25	9	3
Q3THW5	Histone H2A.V	13509	205.61	54	7	5
P0C0S6	Histone H2A.Z	13553	205.61	54	7	5
Q6ZWY9	Histone H2B type 1-C/E/G	13906	165.03	29	5	5
P10853	Histone H2B type 1-F/J/L	13936	165.03	29	5	5
Q64478	Histone H2B type 1-H	13920	165.03	29	5	5
Q8CGP1	Histone H2B type 1-K	13920	165.03	29	5	5
P10854	Histone H2B type 1-M	13936	165.03	29	5	5
Q8CGP2	Histone H2B type 1-P	13992	165.03	29	5	5
Q64525	Histone H2B type 2-B	13920	165.03	29	5	5
Q60960	Importin subunit alpha-5	60183	50.29	7	2	2
Q8K2V6	Importin-11	112416	128.93	7	4	4
Q8BKG3	Inactive tyrosine-protein kinase 7	117532	58.41	3	2	2
O89051	Integral membrane protein 2B	30260	137.73	15	4	4
Q6P4S8	Integrator complex subunit 1	245165	84.79	2	2	2
Q80UK8	Integrator complex subunit 2	133515	79.44	4	2	2
Q7TPD0	Integrator complex subunit 3	117938	177.78	3	5	5
Q62470	Integrin alpha-3	116745	293.5	36	23	23
O55222	Integrin-linked protein kinase	51373	148.78	26	8	8
Q61703	Inter-alpha-trypsin inhibitor heavy chain H2	105928	130.17	5	5	5
Q61704	Inter-alpha-trypsin inhibitor heavy chain H3	99358	121.64	5	5	5
Q00560	Interleukin-6 receptor subunit beta	102451	158.47	10	5	5
Q9QWL7	Keratin type I cytoskeletal 17	48162	157.34	22	10	2
Q6IFZ6	Keratin type II cytoskeletal 1b	61359	128.51	9	5	2
P50446	Keratin type II cytoskeletal 6A	59335	135.97	13	7	2
Q8VED5	Keratin type II cytoskeletal 79	57552	107.43	12	5	2
Q8K274	Ketosamine-3-kinase	34468	119.81	7	2	2
Q60749	KH domain-containing RNA-binding signal transduction-associated protein 1	48371	90.9	8	2	2
Q8C3Y4	Kinetochore-associated protein 1	250356	49.68	2	3	3
Q8BGA5	KRR1 small subunit processome component homolog	43538	54.26	7	2	2
Q61033	Lamina-associated polypeptide 2 isoforms alpha/zeta	75168	99.13	8	3	3

P02468	Laminin subunit gamma-1	177298	265.54	22	20	20
Q6ZQ58	La-related protein 1	121125	97.32	6	3	3
Q8BWW4	La-related protein 4	79763	57.09	3	2	2
O89013	Leptin receptor gene-related protein	14316	67.7	11	2	2
Q9CXD9	Leucine-rich repeat-containing protein 17	51852	118.69	12	4	4
P11152	Lipoprotein lipase	53109	149.06	16	5	5
P16125	L-lactate dehydrogenase B chain	36572	98.39	10	3	2
P35951	Low-density lipoprotein receptor	94947	83.74	5	3	3
P51885	Lumican	38265	112.27	13	4	4
P70699	Lysosomal alpha-glucosidase	106248	78.68	4	2	2
P17047	Lysosome-associated membrane glycoprotein 2	45681	92.42	9	4	4
P58022	Lysyl oxidase homolog 2	87003	121.17	6	3	3
A2AJI0	MAP7 domain-containing protein 1	93276	128.07	9	4	4
Q8K310	Matrin-3	94630	86.88	6	3	3
Q9CQ39	Mediator of RNA polymerase II transcription subunit 21	15588	52.01	41	3	3
Q80YQ2	Mediator of RNA polymerase II transcription subunit 23	156087	90.92	6	4	4
Q9QYH6	Melanoma-associated antigen D1	85670	79.95	4	2	2
Q8K4B0	Metastasis-associated protein MTA1	80798	105.83	7	4	3
Q99LB6	Methionine adenosyltransferase 2 subunit beta	37393	75.93	11	2	2
Q9Z2D8	Methyl-CpG-binding domain protein 3	32168	52.32	6	2	2
Q62000	Mimecan	34012	131.85	18	4	4
Q9JM52	Misshapen-like kinase 1	147294	110.46	4	5	2
Q63844	Mitogen-activated protein kinase 3	43067	143.17	23	6	3
Q9D071	MMS19 nucleotide excision repair protein homolog	113089	72.97	5	3	3
Q9D0I8	mRNA turnover protein 4 homolog	27546	84.41	17	3	3
P21447	Multidrug resistance protein 1A	140646	145.18	4	2	2
Q9R0E1	Multifunctional procollagen lysine hydroxylase and glycosyltransferase LH3	84922	80.69	8	4	4
Q78HU3	Multivesicular body subunit 12A	28705	104.26	12	2	2
Q6KAU4	Multivesicular body subunit 12B	35411	162.79	26	4	4
P28665	Murinoglobulin-1	165297	90.38	3	4	2
P28666	Murinoglobulin-2	162381	90.38	3	4	2
Q6ZPE2	Myotubularin-related protein 5	208691	82.55	3	3	3

Q9DBH0	NEDD4-like E3 ubiquitin-protein ligase WWP2	98761	105.32	5	3	3
Q8K1S3	Netrin receptor UNC5B	103739	102.58	7	4	4
O09118	Netrin-1	67810	153.78	16	6	6
Q04690	Neurofibromin	319595	67	1	2	2
Q9D0T1	NHP2-like protein 1	14174	66.1	31	2	2
O88322	Nidogen-2	153913	274.7	26	22	21
Q80TM9	Nischarin	175011	161.2	5	5	5
Q99ME9	Nucleolar GTP-binding protein 1	74113	80.87	5	3	3
Q9CPT5	Nucleolar protein 16	21139	100.11	19	3	3
Q9D6Z1	Nucleolar protein 56	64464	232.24	29	13	13
Q3V1G4	Olfactomedin-like protein 2B	83503	124.59	10	5	5
B2RUR8	OTU domain-containing protein 7B	91984	78.2	5	2	2
S4R1M9	Oxysterol-binding protein-related protein 10	83856	48.64	3	2	2
Q8CI95	Oxysterol-binding protein-related protein 11	83629	48.64	3	2	2
Q9DCE5	p21-activated protein kinase-interacting protein 1	42116	122.51	16	4	4
P00688	Pancreatic alpha-amylase	57318	37.94	4	2	2
A2AR02	Peptidyl-prolyl cis-trans isomerase G	88325	66.41	2	2	2
Q9R0L6	Pericentriolar material 1 protein	228845	80.73	1	2	2
Q99LL5	Periodic tryptophan protein 1 homolog	55587	74.28	7	2	2
Q3UQ28	Peroxidasin homolog	165102	191.45	11	10	10
P83870	PHD finger-like domain-containing protein 5A	12405	79.66	26	2	2
P70182	Phosphatidylinositol 4-phosphate 5-kinase type-1 alpha	60485	64.1	6	2	2
Q8R2H9	Phosphoethanolamine/phosphocholine phosphatase	29911	50.29	13	2	2
P55065	Phospholipid transfer protein	54453	131.44	18	6	6
P97298	Pigment epithelium-derived factor	46234	152.33	14	4	4
Q68FH0	Plakophilin-4	131551	36.43	2	2	2
P20918	Plasminogen	90808	39.12	2	2	2
Q6PB93	Polypeptide N-acetylgalactosaminyltransferase 2	64515	48.21	5	2	2
Q61838	Pregnancy zone protein	165852	87.77	3	4	2
Q9R1C7	Pre-mRNA-processing factor 40 homolog A	108481	54.81	3	2	2
Q5SWD9	Pre-rRNA-processing protein TSR1 homolog	92105	117.93	9	4	4
Q501J6	Probable ATP-dependent RNA helicase DDX17	72400	208.55	31	17	8

Q9JJY4	Probable ATP-dependent RNA helicase DDX20	91710	168.74	11	5	5
Q569Z5	Probable ATP-dependent RNA helicase DDX46	117448	70.3	3	3	3
Q8BWU5	Probable tRNA N6-adenosine threonylcarbamoyltransferase	36301	98.12	14	2	2
Q61398	Procollagen C-endopeptidase enhancer 1	50168	220.34	49	14	14
Q8R4W6	Procollagen C-endopeptidase enhancer 2	45408	79.43	7	2	2
Q9R0E2	Procollagen-lysine 2-oxoglutarate 5-dioxygenase 1	83595	94.18	7	3	3
Q60716	Prolyl 4-hydroxylase subunit alpha-2	61002	139.84	23	7	7
Q9R0Q7	Prostaglandin E synthase 3	18721	138.42	26	3	3
Q5SSW2	Proteasome activator complex subunit 4	211195	99.24	4	5	5
Q9JK23	Proteasome assembly chaperone 1	33104	58.95	10	2	2
P49722	Proteasome subunit alpha type-2	25927	216.19	43	7	7
O55234	Proteasome subunit beta type-5	28532	223.61	50	11	11
Q91VH6	Protein MEMO1	33692	129.96	29	5	5
P33215	Protein NEDD1	71292	97.2	9	3	3
Q7TSC1	Protein PRRC2A	229200	82.05	4	4	4
Q0VGY8	Protein TANC1	200803	88.92	2	2	2
P19221	Prothrombin	70269	187.94	9	5	5
Q91XY4	Protocadherin gamma-A4	100358	45.89	3	2	2
Q8BKS9	Pumilio homolog 3	72800	124.54	17	6	6
Q68FL4	Putative adenosylhomocysteinase 3	66899	72.7	5	4	2
Q99K01	Pyridoxal-dependent decarboxylase domain-containing protein 1	87336	45.49	4	2	2
P53657	Pyruvate kinase PKLR	62309	70.25	7	3	2
P52480	Pyruvate kinase PKM	57845	369.93	59	30	29
P69566	Ran-binding protein 9	71012	69.77	6	2	2
Q60790	Ras GTPase-activating protein 3	95987	80.86	7	4	4
F6SEU4	Ras/Rap GTPase-activating protein SynGAP	148238	41.66	4	2	2
P35288	Ras-related protein Rab-23	26678	79.2	12	3	3
Q921E2	Ras-related protein Rab-31	21331	75.53	13	2	2
Q64008	Ras-related protein Rab-34	29101	142.28	27	6	5
Q8BU31	Ras-related protein Rap-2c	20745	151.35	40	5	2
P35822	Receptor-type tyrosine-protein phosphatase kappa	164185	81.73	4	4	4
B0V2N1	Receptor-type tyrosine-protein phosphatase S	211902	89.73	2	2	2

Q8VE37	Regulator of chromosome condensation	44931	198.28	23	6	6
Q9EPU0	Regulator of nonsense transcripts 1	123967	184.22	15	12	12
Q8VEE4	Replication protein A 70 kDa DNA-binding subunit	69037	111.93	20	9	9
Q61210	Rho guanine nucleotide exchange factor 1	102804	139.56	8	5	5
Q91VI7	Ribonuclease inhibitor	49817	278.13	50	14	14
O88796	Ribonuclease P protein subunit p30	29473	141.75	29	5	5
P56183	Ribosomal RNA processing protein 1 homolog A	54777	56.34	9	2	2
P97452	Ribosome biogenesis protein BOP1	82546	145.15	16	5	5
Q9JJA4	Ribosome biogenesis protein WDR12	47347	111.22	10	2	2
Q9JJ80	Ribosome production factor 2 homolog	35364	42.48	8	2	2
Q8K224	RNA cytidine acetyltransferase	115418	103.37	4	3	3
B2RY56	RNA-binding protein 25	99552	95.07	7	5	5
Q8C5L7	RNA-binding protein 34	41326	134.46	17	4	4
Q64012	RNA-binding protein Raly	33188	153.09	21	4	4
P35550	rRNA 2'-O-methyltransferase fibrillarin	34307	190.96	46	9	6
Q6P5B0	RRP12-like protein	143131	105.08	3	2	2
Q80SW1	S-adenosylhomocysteine hydrolase-like protein 1	58952	72.7	6	4	2
Q3THS6	S-adenosylmethionine synthase isoform type-2	43689	49.74	10	2	2
Q8K021	Secretory carrier-associated membrane protein 1	38029	37.54	10	2	2
Q62177	Semaphorin-3B	82700	177.73	20	9	9
Q9QZI9	Serine incorporator 3	52623	81.54	5	3	3
Q8BHJ6	Serine incorporator 5	51831	164.43	13	4	4
Q9D6X6	Serine protease 23	43072	155.91	16	4	4
Q9R118	Serine protease HTRA1	51214	276.35	39	16	16
O55098	Serine/threonine-protein kinase 10	111906	40.65	3	2	2
Q7TT50	Serine/threonine-protein kinase MRCK beta	194751	104.08	3	3	3
Q9JLN9	Serine/threonine-protein kinase mTOR	288788	131.38	4	7	7
Q61136	Serine/threonine-protein kinase PRP4 homolog	116976	77.33	3	2	2
Q61151	Serine/threonine-protein phosphatase 2A 56 kDa regulatory subunit epsilon isoform	54714	50.65	6	2	2
Q9CQR6	Serine/threonine-protein phosphatase 6 catalytic subunit	35159	46.99	10	2	2
Q99MR6	Serrate RNA effector molecule homolog	100452	99.86	4	3	3
P07724	Serum albumin	68693	150.13	7	3	3

Q91X20	Set1/Ash2 histone methyltransferase complex subunit ASH2	68250	86.98	5	2	2
Q9R0P3	S-formylglutathione hydrolase	31320	265.07	61	9	9
O89032	SH3 and PX domain-containing protein 2A	124201	49.38	2	2	2
Q8CIF6	SID1 transmembrane family member 2	94501	51.38	5	2	2
Q3TD49	Signal peptide peptidase-like 2B	63824	84.1	7	2	2
Q9D7A6	Signal recognition particle 19 kDa protein	16191	91.47	27	2	2
Q9R1B9	Slit homolog 2 protein	168782	54.54	2	2	2
P27048	Small nuclear ribonucleoprotein-associated protein B	23656	69.8	21	2	2
Q5XG71	Small subunit processome component 20 homolog	317744	78.69	2	3	3
Q9JKZ2	Sodium/myo-inositol cotransporter	79583	54.07	4	2	2
Q61609	Sodium-dependent phosphate transporter 1	74153	92.47	8	4	4
Q8BV57	Soluble scavenger receptor cysteine-rich domain-containing protein SSC5D	144636	98.41	3	3	3
Q9EPR4	Solute carrier family 23 member 2	70049	135.76	8	3	3
Q8VE96	Solute carrier family 35 member F6	40978	70.63	11	2	2
Q8BVL3	Sorting nexin-17	52797	62.51	7	2	2
Q8QZY9	Splicing factor 3B subunit 4	44356	80.62	9	2	2
Q923D4	Splicing factor 3B subunit 5	10119	68.68	33	2	2
P59708	Splicing factor 3B subunit 6	14585	103.43	32	3	3
O70551	SRSF protein kinase 1	73089	127.78	12	5	5
Q8CG48	Structural maintenance of chromosomes protein 2	134239	64.74	4	3	3
Q8CG47	Structural maintenance of chromosomes protein 4	146894	83.13	4	3	3
P70279	Surfeit locus protein 6	41235	53.84	6	2	2
P97801	Survival motor neuron protein	31254	48.2	15	2	2
P97496	SWI/SNF complex subunit SMARCC1	122890	118.05	9	6	4
O54941	SWI/SNF-related matrix-associated actin-dependent regulator of chromatin subfamily E member 1	46638	66.12	10	3	3
Q9EQT6	Synaptotagmin-13	46870	116.9	22	4	4
O88746	Target of Myb protein 1	54325	113.88	17	4	4
P97499	Telomerase protein component 1	291458	130.18	7	10	10
Q80YX1	Tenascin	231805	338.23	26	37	37
Q8CGB6	Tensin-2	152012	112.76	5	4	3
Q3URQ0	Testis-expressed protein 10	105209	89.58	5	3	3
F7BWT7	Tetraspanin-15	33071	113.42	15	3	3

Q9QY33	Tetraspanin-3	28049	68.36	6	2	2
Q9DCK3	Tetraspanin-4	26054	146.69	20	2	2
B1AZI6	THO complex subunit 2	182772	42.76	3	2	2
Q9D0R2	Threonine--tRNA ligase cytoplasmic	83356	120.99	12	6	6
P35441	Thrombospondin-1	129647	123.8	9	8	7
Q03350	Thrombospondin-2	129882	134.72	5	5	4
Q9QZ06	Toll-interacting protein	30345	91.89	10	2	2
O55201	Transcription elongation factor SPT5	120664	84.05	3	2	2
Q6PFR5	Transformer-2 protein homolog alpha	32316	48.47	10	2	2
P62996	Transformer-2 protein homolog beta	33666	159.68	24	5	5
Q62219	Transforming growth factor beta-1-induced transcript 1 protein	50101	49.2	5	2	2
Q8BGN6	Transmembrane gamma-carboxyglutamic acid protein 4	25401	109.42	18	2	2
Q8R138	Transmembrane protein 119	29401	63	15	2	2
Q9QY73	Transmembrane protein 59	36314	122.74	8	2	2
Q8C708	Transmembrane protein C16orf54 homolog	24532	64.78	14	2	2
Q6P2B1	Transportin-3	104170	69.22	4	2	2
Q99PP9	Tripartite motif-containing protein 16	62943	75.39	6	2	2
Q1HFZ0	tRNA (cytosine(34)-C(5))-methyltransferase	85452	71.08	4	2	2
P32921	Tryptophan--tRNA ligase cytoplasmic	54358	172.16	27	9	9
A2AQ07	Tubulin beta-1 chain	50441	169.65	17	9	3
Q7TMM9	Tubulin beta-2A chain	49907	346.94	75	32	2
Q9CR75	Tumor necrosis factor receptor superfamily member 12A	13641	134.68	33	5	5
Q3TWL2	Type 1 phosphatidylinositol 4 5-bisphosphate 4-phosphatase	30047	53.3	8	2	2
Q9CZX7	Type 2 phosphatidylinositol 4 5-bisphosphate 4-phosphatase	28038	226.41	44	8	8
P39688	Tyrosine-protein kinase Fyn	60675	108.34	13	6	2
P52332	Tyrosine-protein kinase JAK1	133367	75.67	4	3	3
Q00993	Tyrosine-protein kinase receptor UFO	98191	85	4	2	2
Q04736	Tyrosine-protein kinase Yes	60630	109.24	17	7	3
Q8C7V3	U3 small nucleolar RNA-associated protein 15 homolog	59375	86.85	11	3	3
Q8R2N2	U3 small nucleolar RNA-associated protein 4 homolog	76909	47.68	5	2	2
Q8CCF0	U4/U6 small nuclear ribonucleoprotein Prp31	55430	45.59	8	2	2
Q9DAW6	U4/U6 small nuclear ribonucleoprotein Prp4	58370	91.46	11	4	4

Q3TIX9	U4/U6.U5 tri-snRNP-associated protein 2	65146	74.82	6	2	2
Q8R5H1	Ubiquitin carboxyl-terminal hydrolase 15	112325	59.01	5	4	4
Q6A4J8	Ubiquitin carboxyl-terminal hydrolase 7	128475	76.03	3	2	2
Q9ES00	Ubiquitin conjugation factor E4 B	133317	71.64	2	2	2
Q91WB7	Ubiquitin domain-containing protein 1	25985	51.88	21	2	2
Q8CGY8	UDP-N-acetylglucosamine--peptide N-acetylglucosaminyltransferase 110 kDa subunit	116952	89.14	7	5	5
Q9CXL3	Uncharacterized protein C7orf50 homolog	22168	83.66	22	2	2
E9Q634	Unconventional myosin-Ie	126818	148.94	9	6	5
Q99104	Unconventional myosin-Va	215536	100.21	2	3	3
Q9JMH9	Unconventional myosin-XVIIIa	232753	59.2	2	3	3
P54728	UV excision repair protein RAD23 homolog B	43513	85.78	9	2	2
Q8R0J7	Vacuolar protein sorting-associated protein 37B	31056	190.8	36	6	6
Q8R105	Vacuolar protein sorting-associated protein 37C	38453	126.59	14	3	3
P46467	Vacuolar protein sorting-associated protein 4B	49419	165.1	23	7	7
Q9CQ80	Vacuolar protein-sorting-associated protein 25	20748	80.34	23	4	4
Q62059	Versican core protein	366788	243.81	7	16	16
P70280	Vesicle-associated membrane protein 7	24967	79.16	22	3	3
P63082	V-type proton ATPase 16 kDa proteolipid subunit	15808	130.57	54	3	3
Q9CR51	V-type proton ATPase subunit G 1	13724	152.11	26	3	3
Q8K1X1	WD repeat-containing protein 11	135937	80.41	5	3	3
Q8C6G8	WD repeat-containing protein 26	70544	60.43	5	2	2
Q6ZQL4	WD repeat-containing protein 43	75381	82.57	7	2	2
Q99ME2	WD repeat-containing protein 6	121898	40.23	5	2	2
Q3U821	WD repeat-containing protein 75	94037	71.37	4	2	2
Q8BFQ4	WD repeat-containing protein 82	35079	119.64	11	2	2
