# Supplementary figures and images for: Physiactisome: A New Nanovesicle Drug Containing Heat Shock Protein 60 for Treating Muscle Wasting and Cachexia
Source: Cells. 2022 Apr 21;11(9):1406. doi: 10.3390/cells11091406 (PMC9100106; doi:10.3390/cells11091406)

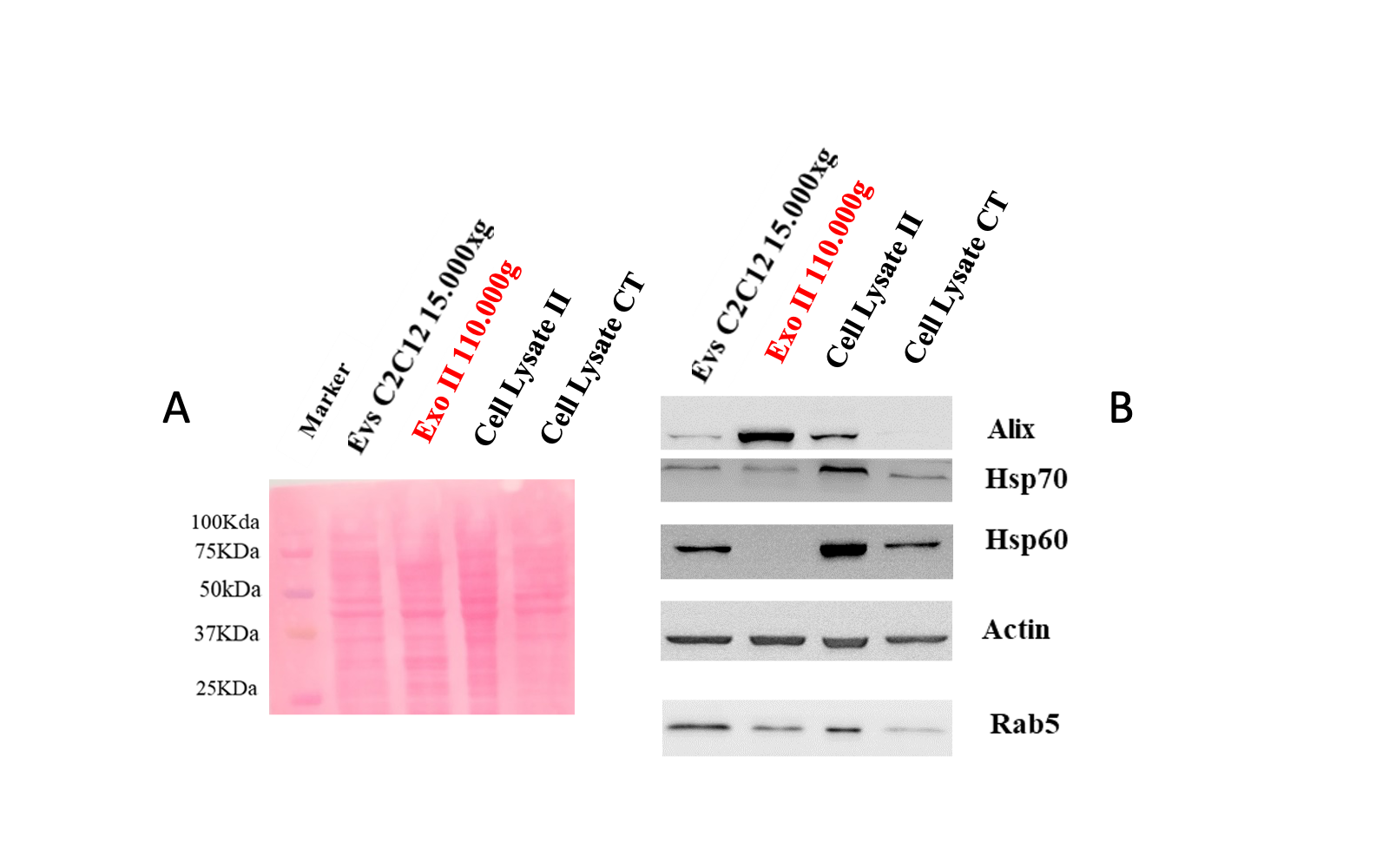

Supplement: Supplementary file 1 [file cells-11-01406-s001.zip › Supporting Information Figure S1.tiff]

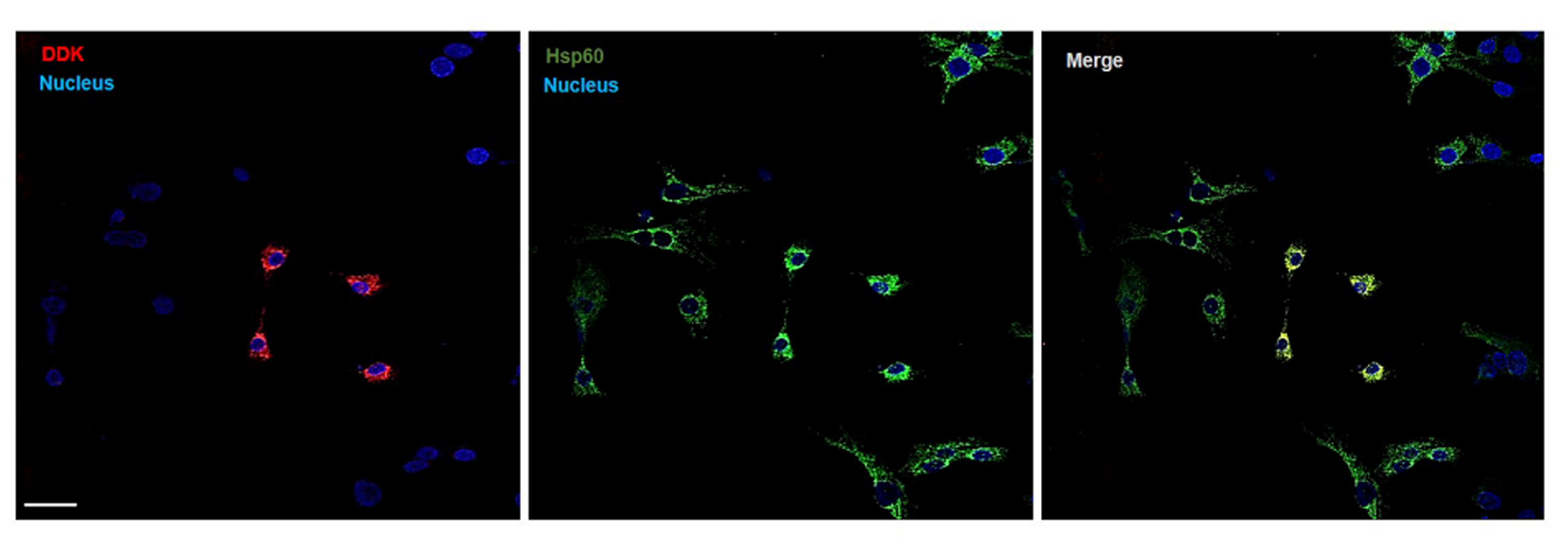

Supplement: Supplementary file 1 [file cells-11-01406-s001.zip › Supporting Information Figure S2.tif]
